# Disturbance of cardiac gene expression and cardiomyocyte structure predisposes *Mecp2*-null mice to arrhythmias

**DOI:** 10.1038/srep11204

**Published:** 2015-06-15

**Authors:** Munetsugu Hara, Tomoyuki Takahashi, Chiaki Mitsumasu, Sachiyo Igata, Makoto Takano, Tomoko Minami, Hideo Yasukawa, Satoko Okayama, Keiichiro Nakamura, Yasunori Okabe, Eiichiro Tanaka, Genzou Takemura, Ken-ichiro Kosai, Yushiro Yamashita, Toyojiro Matsuishi

**Affiliations:** 1 Division of Gene Therapy and Regenerative Medicine, Cognitive and Molecular Research Institute of Brain Diseases; 2 Cardiovascular Research Institute, Kurume University, Kurume, Japan; 3 Department of Pediatrics, Kurume University School of Medicine, Kurume, Japan; 4 Department of Physiology, Kurume University School of Medicine, Kurume, Japan; 5 Department of Anatomy, Kurume University School of Medicine, Kurume, Japan; 6 Department of Internal Medicine, Asahi University School of Dentistry, Gifu, Japan; 7 Department of Gene Therapy and Regenerative Medicine, Advanced Therapeutics Course, Kagoshima University Graduate School of Medical and Dental Sciences, Kagoshima, Japan

## Abstract

Methyl-CpG-binding protein 2 (MeCP2) is an epigenetic regulator of gene expression that is essential for normal brain development. Mutations in MeCP2 lead to disrupted neuronal function and can cause Rett syndrome (RTT), a neurodevelopmental disorder. Previous studies reported cardiac dysfunction, including arrhythmias in both RTT patients and animal models of RTT. In addition, recent studies indicate that MeCP2 may be involved in cardiac development and dysfunction, but its role in the developing and adult heart remains unknown. In this study, we found that Mecp2-null ESCs could differentiate into cardiomyocytes, but the development and further differentiation of cardiovascular progenitors were significantly affected in MeCP2 deficiency. In addition, we revealed that loss of MeCP2 led to dysregulation of endogenous cardiac genes and myocardial structural alterations, although Mecp2-null mice did not exhibit obvious cardiac functional abnormalities. Furthermore, we detected methylation of the CpG islands in the Tbx5 locus, and showed that MeCP2 could target these sequences. Taken together, these results suggest that MeCP2 is an important regulator of the gene-expression program responsible for maintaining normal cardiac development and cardiomyocyte structure.

Methyl-CpG-binding protein 2 (MeCP2) plays a critical role in regulating chromatin conformation and epigenetic gene expression through a methyl-CpG-binding domain and a transcriptional repression domain^1^,^2^,^3^. MeCP2 acts as both a repressor and an activator to control the expression of various genes via recruitment of chromatin remodeling complexes such as Sin3a, histone deacetylase (HDAC) 1/2, nuclear receptor corepressor (N-CoR) / silencing mediator for retinoid and thyroid hormone receptors (SMRT), RE1-silencing transcription factor (REST) / neuron-restrictive silencer factor (NRSF), suppressor of variegation 3–9 homolog 1 (Suv39H1), histone methyltransferase, and DNA methyltransferase I^1^,^2^. Although MeCP2 is expressed in several mouse tissues including brain, lung, skeletal muscle, and heart, its relevance to neuronal function became evident only after the finding that mutations in the MeCP2 gene cause Rett syndrome (RTT)^4^,^5^,^6^.

RTT (MIM #312750) is a neurodevelopmental disorder with a high female gender bias, affecting roughly 1 in 10,000 live female births. The vast majority (90–95%) of typical RTT cases harbor a loss-of-function mutation in the X-linked gene encoding MeCP2^2^,^6^,^7^. Knockout mouse models with disrupted MeCP2 function mimic many key clinical features of RTT, including normal early postnatal life followed by developmental regression resulting in motor impairment, hindlimb clasping, irregular breathing, and cardiac abnormalities^3^,^8^,^9^.

One of the most unfortunate features of RTT is the associated mortality rate of 1.2% per year; of those deaths, 26% are sudden and unexpected^10^,^11^,^12^,^13^. The pathogenesis of sudden death in RTT is unknown, but is strongly suspected to involve cardiac dysfunction. Previous studies found prolongation of the corrected QT interval and lethal cardiac arrhythmias in both RTT patients and animal models^10^,^12^. Several studies have suggested that cardiac dysfunction in RTT may be secondary to abnormal nervous system control^14^,^15^,^16^,^17^. However, accumulating evidence indicates that the cardiac dysfunction observed in RTT may also result from MeCP2 deficiency in the cardiovascular system itself^18^. Recent studies have elucidated the role of MeCP2 in cardiovascular development and cardiomyocyte maturation. Specifically, MeCP2 is expressed in the developing heart, and overexpression of MeCP2 in the heart causes embryonic lethality with cardiac septum hypertrophy^19^. In addition, DNA methylation plays a key role in cardiomyocyte differentiation^20^; MeCP2 is upregulated in differentiated cardiomyocytes, and overexpression of MeCP2 results in an alteration of methylation levels. Although the significance of cardiac expression of MeCP2 is unknown, these studies suggest that MeCP2 may play a functional role in cardiovascular development and physiological function.

In this study, we investigated the contribution of MeCP2 to cardiac development, structure, and function using an *in vitro* ES cell model system and an *in vivo* mouse model for RTT. Our results demonstrate that MeCP2 affects cardiovascular development of ES cell–derived cardiovascular progenitor cells. We also show that MeCP2 is involved in maintaining normal cardiac gene expression and cardiomyocyte structure in the adult mouse heart.

## Results

### Cardiac development of Mecp2-null ES cells

**Figure 1:**
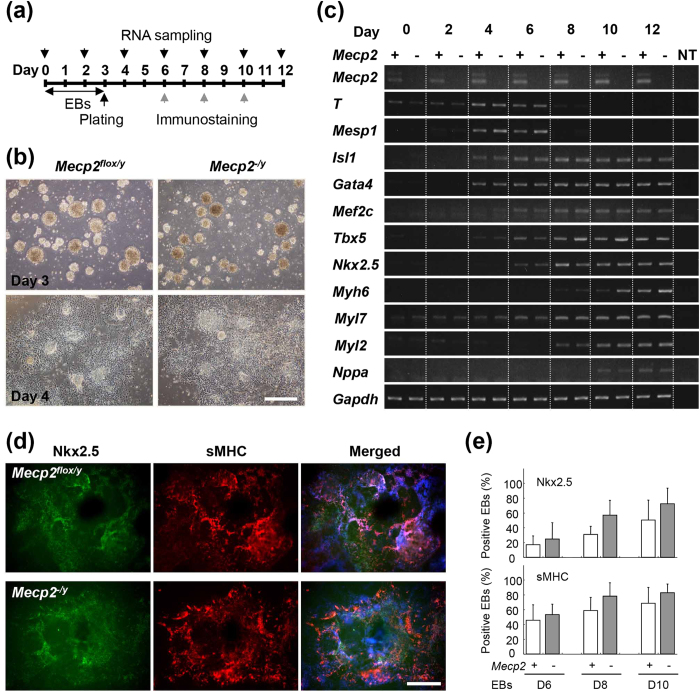
Cardiac differentiation of wild-type and *Mecp2*-null ES cells. (**a**) Experimental schedule. ES cells were cultured in suspension for 3 days to form EBs, plated on gelatin-coated dishes in differentiation medium, and then incubated for 9 days. Differentiation of cardiomyocytes was evaluated by RT-PCR (black arrows) and/or immunostaining (gray arrows) on the indicated days. (**b**) Representative images of wild-type and *Mecp2*-null EBs on the indicated days after EB formation. Scale bar indicates 500 μm. (**c**) Expression of endogenous differentiation markers during ES cell differentiation. Marker gene expression in the wild-type and *Mecp2*-null EBs on the indicated days after EBs formation was analyzed by RT-PCR. (**d**) Cardiac differentiation of wild-type and *Mecp2*-null ES cells. Wild-type and *Mecp2*-null EBs on day 10 were immunostained with antibodies against Nkx2.5 (green) and sMHC (red). Scale bars indicate 500 μm. (**e**) Percentage of Nkx2.5- or sMHC-positive EBs in the wild-type (white column) and *Mecp2*-null (gray column) cultures on the indicated days. Bars represent means ± SD from six independent experiments.

We first examined the role of MeCP2 during ES cell (ESC) differentiation by comparing the phenotypes of *Mecp2*-null (*Mecp2*^*−/y*^) and wild-type (*Mecp2*^*flox/y*^) ESCs (Fig. 1a). Neither the size nor the morphology of the differentiating embryoid bodies (EBs) differed significantly between the *Mecp2*-null and wild-type groups, and beating EBs were apparent on day 8 in both groups (Fig. 1b).

Reverse-transcription polymerase chain reaction (RT-PCR) analysis revealed that both *Mecp2* variants, e1 and e2, were expressed in wild-type ESCs in the undifferentiated state, as well as throughout all development stages (Fig. 1c). In both *Mecp2*-null and wild-type EBs, *T* (also known as *Bra*) and *Mesp1* began to be expressed at day 4, and then were rapidly downregulated on day 8. Cardiac transcription factors, including the early-stage markers *Isl1*, *Gata4*, *Mef2c*, *Tbx5*,> and *Nkx2.5*,> began to be expressed on days 4 to 6, peaking around day 6. Cardiac structural/physiological genes, such as *Myh6*, *Myl7*, *Myl2*, and *Nppa*, began to be expressed on days 6 to 10, and their expression was maintained thereafter. These results suggest that loss of MeCP2 dose not affect the induction of cardiac marker genes during ESC differentiation.

Next, we investigated whether MeCP2 affected cardiac differentiation of ESCs by immunostaining for Nkx2.5 and sMHC, which are markers for early and differentiated cardiomyocytes, respectively (Fig. 1d,e). In both *Mecp2*-null and wild-type EBs, the percentage of *Nkx2.5*-, and sMHC-positive EBs gradually increased, reaching a maximum 10 days after induction. The percentage of *Nkx2.5*- and sMHC-positive *Mecp2*-null EBs was higher than that of the wild-type EBs on days 8 and 10, although the difference was not statistically significant. These results suggest that MeCP2 is not essential for induction of cardiac differentiation.

### Characterization of cardiovascular progenitor cells derived from Mecp2-null ES cells

**Figure 2:**
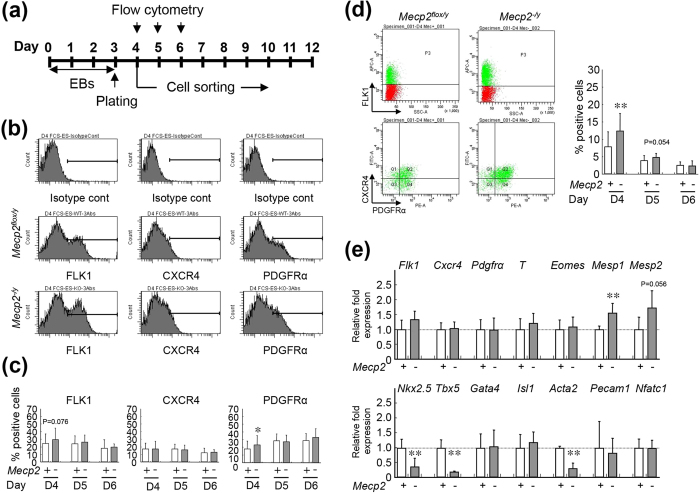
Isolation and characterization of wild-type and *Mecp2*-null ES-derived cardiovascular progenitors. (**a**) Experimental schedule. ES cells were cultured in suspension for 3 days to form EBs, and then plated on gelatin-coated dishes. Flow-cytometric analysis and sorting of FLK1/CXCR4/PDGFRα-positive cardiovascular progenitors were performed on days 4, 5, and 6 after EB formation. (**b**) Detection of FLK1-, CXCR4-, or PDGFRα-positive cells in wild-type and *Mecp2*-null EBs by flow cytometry on day 4 after EB induction. (**c**) Percentages of FLK1 (left), CXCR4 (middle), or PDGFRα (right) positive cells in wild-type (white column; days 4, 5, and 6; n = 49, 17, and 11, respectively) and *Mecp2*-null (gray column; days 4, 5, and 6; n = 50, 17, and 11, respectively) EBs on days 4, 5, and 6. Bars represent the mean ± SD from at least 10 independent experiments (^*^p < 0.05). (**d**) Detection of FLK1/CXCR4/PDGFRα-positive cells by flow cytometry on day 4 after induction of wild-type (left) and *Mecp2*-null (right) EBs. The graphs show the percentage of FLK1/CXCR4/PDGFRα–positive progenitors in wild-type (white column) and *Mecp2*-null (gray column) EBs on days 4 (n = 40), 5 (n = 16), and 6 (n = 10). Bars represent the means ± SD from at least 10 independent experiments (^**^p < 0.01). (**e**) Marker gene expression in isolated progenitors on day 4 after induction of EBs. Expression levels of marker genes was measured by qRT-PCR in wild-type (white column) and *Mecp2*-null (gray column) isolated cells, and normalized against *Gapdh*. Each *Gapdh*-normalized mRNA level was further normalized against the corresponding mRNA level in the wild-type group. Data are shown as fold change relative to the wild-type group (defined as 1.0). Data are represented as means ± SD from five independent experiments (^**^p < 0.01).

Several recent studies used flow cytometry to characterize the cardiac stem/progenitor cells from EBs, and demonstrated that FLK1, CXCR4, and PDGFRα are feasible markers for detection of multipotent cardiovascular progenitor cells (CPCs)^21^,^22^,^23^. To confirm the effects of MeCP2 deficiency on cardiac development, we isolated CPCs derived from wild-type and *Mecp2*-null EBs (Fig. 2a).

RT-PCR analysis revealed that undifferentiated *Mecp2*-null ESCs did not express *Flk1* and *Pdgfra* mRNA, but did express *Cxcr4* at levels comparable to those in wild-type ESCs. During EB differentiation, *Flk1* and *Pdgfra* began to be expressed on day 4, and their expression was maintained thereafter. We also observed prominent expression of *Cxcr4* on day 4 after ESC differentiation in both strains (data not shown). Therefore, we used flow cytometry to evaluate the percentage of cells positive for these cardiac progenitor markers on days 4–6 of EB culture (Fig. 2b,c). In both *Mecp2*-null and wild-type EBs, *FLK1*-, CXCR4-, or PDGFRα-positive cells could be detected during this period, and FLK1- or CXCR4-positive cells were present at the highest levels on day 4. The proportion of PDGFRα-positive cells gradually increased during EB differentiation in cultures from both strains. The percentages of CXCR4-positive cells did not change significantly, whereas the percentage of PDGFRα-positive cells was higher in *Mecp2*-null EBs than in wild-type EBs on day 4. The percentage of FLK1-positive cells was also higher in the *Mecp2*-null group, but not significantly. In addition, FLK1-, CXCR4-, and PDGFRα- triple-positive (FLK1^+^/CXCR4^+^/PDGFRα^+^-) CPCs were present at the highest level on day 4, but the abundance of these cells rapidly decreased thereafter (Fig. 2d). Although the temporal profiles of marker expression were grossly similar in both strains, *Mecp2*-null EBs contained a higher percentage of FLK1^+^/CXCR4^+^/PDGFRα^+^ CPCs than wild-type EBs on day 4. Thus, cardiac progenitors were developed at higher levels in *Mecp2*-null cultures than in control cultures. These results suggest that MeCP2 deficiency affected the CPC development during ESC differentiation.

### Transcriptional profiling of cardiovascular progenitor cells derived from Mecp2-null ES cells

The highest percentages of FLK1^+^/CXCR4^+^/PDGFRα^+^ CPCs were detected in both *Mecp2*-null and wild-type EBs on day 4. To compare the early molecular events occurring in *Mecp2*-null EBs with those in wild-type EBs, we isolated FLK1^+^/CXCR4^+^/PDGFRα^+^ CPCs by FACS on day 4, and then investigated the transcriptional profiles of both groups by DNA microarray analysis. In particular, we determined which genes exhibited a change in expression of *≥*2.0-fold between *Mecp2*-null and wild-type sorted CPCs on day 4 after EBs differentiation. Using these criteria, we found that 2,296 probes out of 62,969 were differentially expressed between *Mecp2*-null and wild-type cardiovascular progenitors. In the *Mecp2*-null CPCs, 1,236 probes were upregulated, whereas 1,060 probes were downregulated. A number of genes with the potential to affect cardiac development were highly dysregulated in *Mecp2*-null CPCs (Supplementary Table S1). Downregulated transcripts included *Acta2*, *Tbx5*, *Lefty1*, *Nkx2.5*, *Myocd*, and *Nog*, which are essential for heart development. Upregulated genes included *T2* (also known as *Bra2*), *Mesp2*, *Lif*, and *Socs2*,> which play crucial roles in early mesoderm development. Several of the differentially expressed genes are of unknown function (data not shown). The list of cardiovascular genes is available in supporting online material (Supplementary Table S1).

To validate the changes of gene expression observed in our microarray experiments, we performed quantitative real-time reverse-transcription polymerase chain reaction (qRT-PCR) on some candidate genes using CPCs sorted from independent sets of *Mecp2*-null and wild-type EBs harvested on day 4 (Fig. 2e). For this validation, we chose three genes (*Tbx5*, *Nkx2.5*, *Acta2*) that were significantly down-regulated and two genes (*Mesp1*, *Mesp2*) that were significantly up-regulated in *Mecp2*-null CPCs. We observed a good correlation between microarray results and qRT-PCR data for all five genes evaluated. However, no differences in the expression of *Flk1*, *Cxcr4*, *Pdgfra*, *T*, *Eomes*, *Gata4*, *Isl1*, *Pecam1*, and *Nfatc1*, which play essential roles in early mesoderm or/and cardiovascular development, were detected between the two CPC groups. These data reinforced the notion that MeCP2 deficiency impaired the development and cardiac differentiation potency for CPCs during ESC differentiation.

### Cardiac differentiation of cardiac progenitors derived from Mecp2-null ES cells

**Figure 3:**
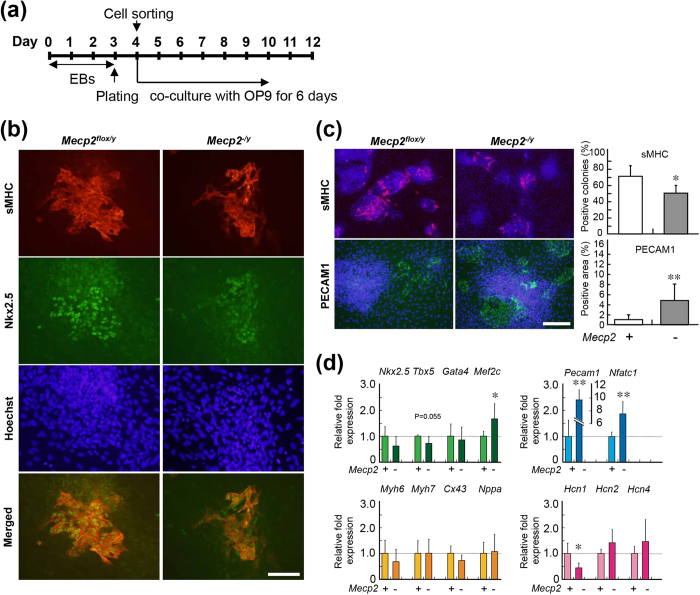
Differentiation of wild-type and *Mecp2*-null cardiovascular progenitors. (**a**) Experimental schedule. On day 4, FLK1/CXCR4/PDGFRα-positive cells obtained from FACS were plated onto OP9 stromal cells to induce differentiation. Differentiation of cardiomyocytes and endothelial cells was evaluated by immunostaining and qRT-PCR on day 10. (**b**) Cardiac differentiation was evaluated by immunostaining for sMHC (red) and Nkx2.5 (green). Scale bar indicates 200 μ m. (**c**) Cardiac and endothelial differentiation were quantified by immunostaining for sMHC (red) and the endothelial marker PECAM1 (green). Nuclei were counterstained with Hoechst 33343 (blue). Scale bar indicates 500 μm. The graphs on the right show the percentage of sMHC-positive colonies (n = 6) or PECAM1-positive cell surface area (n = 12) in the wild-type (white column) and *Mecp2*-null (gray column) cultures. Bars represent the means ± SD from at least 100 colonies measured (^*^p < 0.05, ^**^p < 0.01). (**d**) qRT-PCR analysis of cardiovascular marker genes during differentiation of control (light color column) and *Mecp2*-null (deep color column) progenitors. Each mRNA level was normalized against the level of *Gapdh* mRNA, and the resultant values were further normalized against the corresponding value from non-treated wild-type cultures. Data are shown as fold change relative to wild-type cultures (defined as 1.0). Bars represent the means ± SD from six independent experiments (^*^p < 0.05, ^**^p < 0.01).

To compare the differentiation potency of cardiovascular progenitors in *Mecp2*-null and wild-type EBs, we cultured isolated FLK1^+^/CXCR4^+^/PDGFRα^+^ CPCs on OP9 stromal cells for an additional 6 days, and then subjected these cells to immunostaining for sMHC and PECAM1 (Fig. 3). The percentages of sMHC-positive colonies were significantly lower in *Mecp2*-null CPCs than in wild-type EBs (Fig. 3c). By contrast, the percentages of PECAM1-positive areas were significantly higher in *Mecp2*-null CPCs.

We also used qRT-PCR to examine the change in cardiovascular gene expression between *Mecp2*-null and wild-type CPC cultures (Fig. 3d). Expression of the cardiac markers *Gata4*, *Myh6*, *Myh7*, and *Nppa* in CPC cultures did not differ significantly between the two strains. Expression of *Tbx5*, *Nkx2.5*, and *Cx43* was lower, whereas expression of *Mef2c* was significantly higher, in *Mecp2*-null CPC cultures. Because the expression of cardiac marker genes such as *Tbx5* is mainly dependent on the efficiency of cardiac differentiation of ESCs, we realized that the low expression of *Tbx5* during cardiac differentiation of *Mecp2*-null ESCs might be caused by inefficient cardiac differentiation. We also found that expression of genes related to cardiovascular fate, including the endothelial marker *Pecam1* and the endocardial marker *Nfatc1*, was significantly elevated in *Mecp2*-null cultures, whereas expression of *Hcn1*, a cardiac pacemaker channel, was significantly reduced. These results suggest that MeCP2 deficiency impaired cardiac differentiation of the FLK1^+^/CXCR4^+^/PDGFRα^+^ CPCs. Thus, MeCP2 is not essential for induction of cardiac differentiation, but contributes to the development and further differentiation of cardiovascular progenitors during ESCs differentiation.

### Effects of MeCP2 deficiency on adult heart

**Figure 4:**
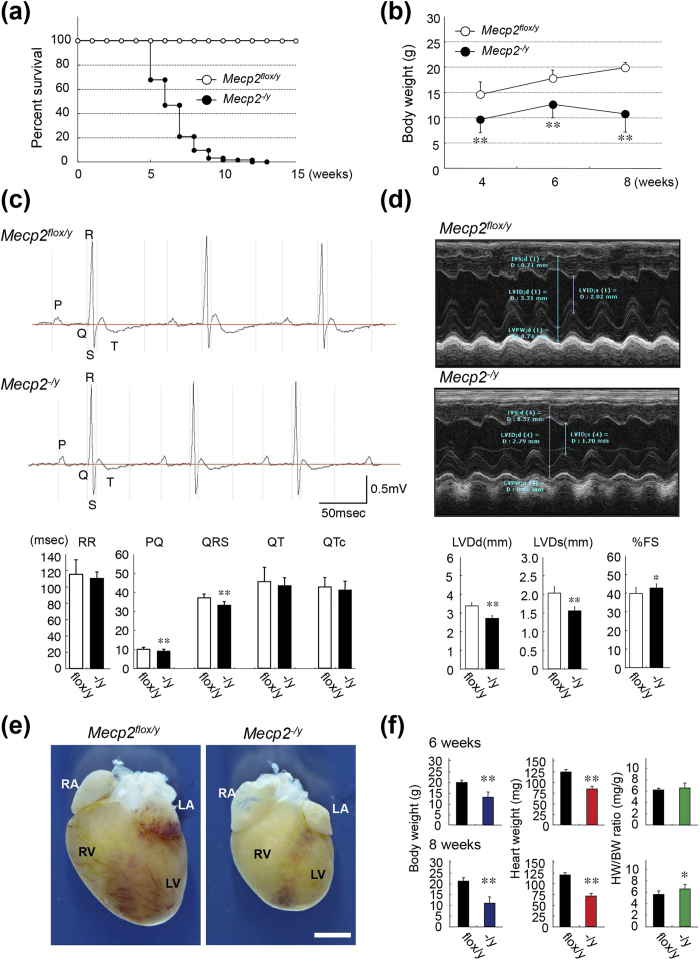
Phenotypic characterization of *Mecp2*-null mice. (**a**) Kaplan–Meier survival curves for *Mecp2*-null mice. Litters including wild-type (*Mecp2*^*flox/y*^) and *Mecp2*-null (*Mecp2*^*−/y*^) pups were followed for 4–15 weeks after birth. Open circles, control wild-type mice (n = 30); closed circles, *Mecp2*-null mice (n = 62). Survival rates differed significantly between wild-type and *Mecp2*-null mice (p < 0.01, long-rank test). (**b**) Body weights in wild-type and *Mecp2*-null mice measured at the indicated times. Data are expressed as means ± SD. ^**^p < 0.01, wild-type (4, 6, and 8-week-old; n = 12, 5, and 12) versus *Mecp2*-null (4, 6, and 8-week-old; n = 87, 82, and 61) mice. **(c)** Representative surface ECG from wild-type and *Mecp2*-null mice at 8 weeks. Graphs show mean time of RR, PQ, QRS, QT, and QTc intervals in 8-week-old wild-type and *Mecp2*-null mice. Data are expressed as means ± SD. ^*^p < 0.05 and ^**^p < 0.01, wild-type versus *Mecp2*-null mice. Mean values of electrocardiographic parameters are shown in Supplementary Table S2. (**d**) Representative M-mode echocardiographic tracings from 8-week-old wild-type and *Mecp2*-null mice. Graphs show mean values of LV end-systolic (LVDs) and -diastolic (LVDd) internal dimension and fraction shortening (%FS) in 8-week-old wild-type and *Mecp2*-null mice. Data are expressed as means ± SD. ^*^p < 0.05 and ^**^p < 0.01, wild-type versus *Mecp2*-null mice. Mean values of echocardiographic parameters are shown in Supplementary Table S3. (**e**) Hearts of wild-type and *Mecp2*-null mice at 8 weeks. Scale bars indicate 2.0 mm. (**f**) Body weights (g), heart weights (mg), and ratios of heart to body weight (mg/g) in wild-type (black column; 6- and 8-week-old; n = 7 and 7) and *Mecp2*-null (colored column; 6- and 8-week-old; n = 7 and 9) mice measured at 6 (top three graphs) or 8 (bottom three graphs) weeks. Data are expressed as means ± SD. ^*^p < 0.05 and ^**^p < 0.01, wild-type versus *Mecp2*-null mice.

Several previous studies reported cardiac abnormalities in both RTT patients and animal models^17^,^18^. In our laboratory, *Mecp2*-null mice exhibited reduced spontaneous movement between 3 and 8 weeks of age. In addition, *Mecp2*-null mice were already substantially underweight at 4 weeks, and died at approximately 10 weeks (Fig. 4a,b). These findings are consistent with the major phenotypes arising from MeCP2 deficiency described in previous reports^9^. Although those *in vivo* studies explored the hypothesis that brain function in *Mecp2*-null mice might decline steeply, leading to early symptoms and death, no firm conclusions have yet been reached^3^.

When we subjected *Mecp2*-null mice to surface electrocardiographic (ECG) analysis, we were unable to detect exhibited abnormal ECG patterns, such as a prolonged QT interval, at the age of 6 or 8 weeks (Fig. 4c). Though a trend was observed toward shorter RR intervals, the RR interval did not significantly differ between wild-type and *Mecp2*-null mice. The QRS and PQ intervals in *Mecp2*-null mice were shorter than those in wild-type mice (Supplementary Table S2). Two-dimensionally targeted M-mode echocardiographic (ECO) analysis, performed at the age of 8 weeks, revealed that LVDd and LVDs in the *Mecp2*-null group were significantly lower than those in wild-type mice, although %FS was slightly higher in *Mecp2*-null mice (Fig. 4d, Supplementary Table S3). We realized that this difference in LV cavity diameter volume was caused by a difference in the size of the heart. In addition, the slight increase in %FS within the normal range in *Mecp2*-null mice might have been influenced by the heart rate (short RR interval). These findings indicate that the macroscopic structures and function of the heart are generally maintained in *Mecp2*-null mice hearts.

### Effects of MeCP2 deficiency on cardiac structures

At the ages of both 6 and 8 weeks, body and heart weights were significantly lower in *Mecp2*-null mice than in wild-type mice (Fig. 4e,f). The ratio of heart to body weight did not differ significantly between *Mecp2*-null and wild-type mice at 6 weeks, but at 8 weeks the ratio in *Mecp2*-null mice was 1.05-fold higher than that in wild-type mice, possibly due to rapid weight loss in the mutant mice. Autopsy failed to reveal any obvious alterations, other than reduced heart size and weight, in *Mecp2*-null mice.

**Figure 5:**
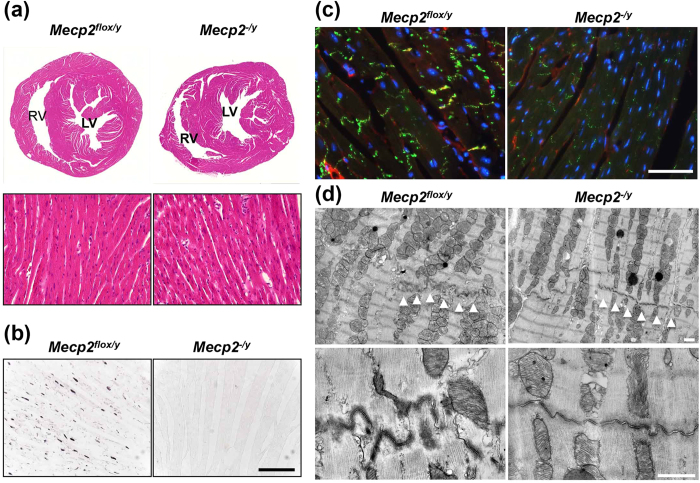
Histological analysis of hearts from *Mecp2*-null mice. (**a**) Representative hematoxylin/eosin-stained transverse sections through hearts of 8-week-old wild-type (left panel) and *Mecp2*-null (right panel) mice. Left and right ventricles are shown. No gross differences were detected in cardiomyocyte structure. Deletion of MeCP2 dose not cause heart defects. (**b**) MeCP2 expression by immunohistochemistry. MeCP2 signals were detected in wild-type left ventricular tissues. Scale bars indicate 100 μm. (**c**) Representative immunofluorescence images showing the distribution of Cx43 signal in wild-type and *Mecp2*-null left ventricular tissues. Cryosections of left ventricular tissue obtained from 8-week-old wild-type (left panel) and *Mecp2*-null (right panel) mice were immunostained for Cx43 (green) and N-cadherin (red), and counterstained with Hoechst 33342 (blue, merged image panels). Alteration in the pattern of Cx43 staining in *Mecp2*-null hearts is evident in comparison with wild-type hearts. Scale bars indicate 50 μm. (**d**) Transmission electron microscopy analysis of left ventricular tissues in wild-type (left panels) and *Mecp2*-null (right panels) mice hearts. Representative electron micrographs show altered IDs morphology in *Mecp2*-null hearts; the IDs (white arrowheads) between cardiomyocytes from *Mecp2*-null mice were less convoluted and contained fainter desmosomes. Scale bars indicate 1.0 μm.

Following hematoxylin and eosin staining, *Mecp2*-null hearts appeared to be histologically normal (Fig. 5). However, immunofluorescence staining of cell–cell junction components, including connexin 43 (Cx43) and N-cadherin, revealed lower levels of both proteins in *Mecp2-n*ull ventricle (Fig. 5c and Supplementary Fig. S1). Electron microscopic examination revealed that intercalated discs (IDs) contained very immature desmosomes and were insufficiently convoluted, thus appearing less developed in *Mecp2*-null mice (Fig. 5d). No structural alterations were observed in sarcomeres and mitochondria from *Mecp2*-null hearts, relative to control hearts. However, nuclei were more remarkably hypertrophied and autophagic vacuoles were more abundant in contrast to fewer lysosomes in *Mecp2*-null cardiomyocytes (Supplementary Fig. S2a). We also found an increase in the LC3-II/LC3-I ratio, which is an established indicator of autophagic turnover (Supplementary Fig. S2b and c). These results suggest that MeCP2 deficiency causes alteration in myocardial ultrastructures and promotes autophagic activity.

### Alterations of endogenous gene expression in MeCP2-deficient heart

**Figure 6:**
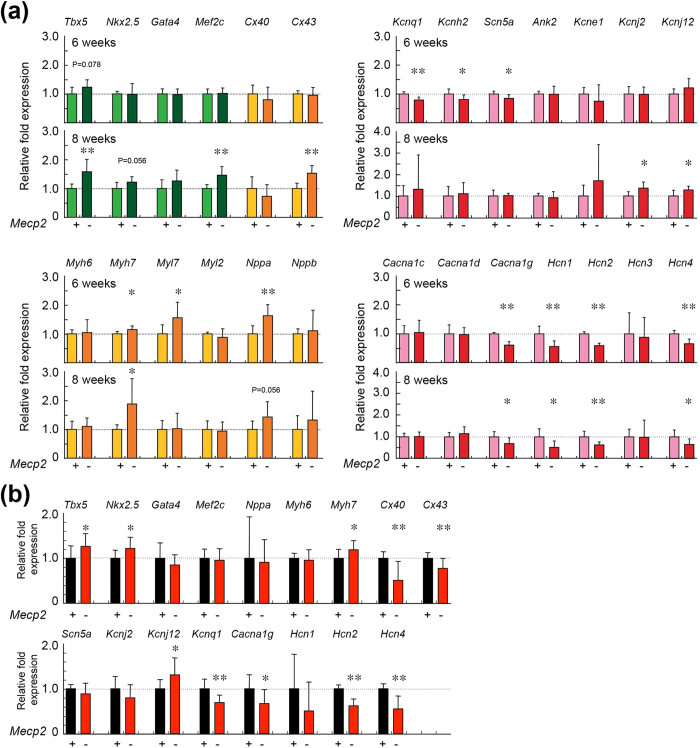
Cardiac gene expression in wild-type and *Mecp2*-null mice hearts. (**a**) Whole hearts in 6- and 8-week-old wild-type and *Mecp2*-null mice was harvested, and qRT-PCR was performed using primers for the indicated genes. The graphs show the relative fold changes of the indicated genes’ mRNA in wild-type (light color columns) and *Mecp2*-null (deep color columns) hearts at 6 (upper graph; wild-type and *Mecp2*-null group; n = 7 and 9) and 8 (lower graph; wild-type and *Mecp2*-null group; n = 7 and 10) weeks of age. (**b**) Ventricular tissues in 8-week-old wild-type and *Mecp2*-null mice were harvested, and qRT-PCR was performed using primers for the indicated genes. The graphs show the relative fold changes of the indicated genes’ mRNA in wild-type (black column; n = 9) and *Mecp2*-null (red column; n = 17) ventricular tissues at 8 weeks of age. The level of each mRNA was normalized to the level of *Gapdh*. All data were normalized to the value from wild-type mice of the same age, which was defined as 1.0. Data are expressed as means ± SD. ^*^p < 0.05, ^**^p < 0.01 versus wild-type mouse samples.

To determine the role of MeCP2 in the regulation of cardiac gene expression *in vivo*,> we performed qRT-PCR analyses of cardiac genes, using total RNA prepared from whole heart taken from 6- or 8-week-old mice (Fig. 6a). At the age of 6 weeks, the mRNA levels of *Myh7, Myl7, and Nppa* were significantly higher in *Mecp2*-null mice than in wild-type mice. In addition, *Tbx5* mRNA levels were higher in *Mecp2*-null mice, although the difference was not statistically significant. At the age of 8 weeks, the mRNA levels of *Tbx5, Mef2c, and Myh7* were significantly higher in the *Mecp2*-null hearts. At 8 weeks, *Nppa* mRNA levels in *Mecp2*- null hearts were higher (although not statistically significantly) than in wild-type hearts. By contrast, *Cx43* mRNA levels were significantly higher in *Mecp2*-null hearts than in wild-type hearts. These results indicate that loss of MeCP2 leads, either directly or indirectly, to transcriptional dysregulation of these cardiac genes in the adult mouse heart.

We next investigated cardiac expression of genes related to voltage-gated channels (Fig. 6a). At both 6 and 8 weeks, *Hcn1*, *Hcn2*, *Hcn4*, and *Cacna1g* mRNA levels were significantly lower in *Mecp2*-null mice than in wild-type mice. As with other *Hcns*, mRNA levels of *Hcn3* in *Mecp2*-null hearts were lower, but not statistically significantly, than those in wild-type hearts, at both 6 and 8 weeks. At 6 weeks, *Kcnq1*, *Kcnh2*, and *Scn5a* mRNA levels were significantly lower in the hearts of *Mecp2*-null mice. By contrast, *Kcnj2* and *Kcnj12* mRNA levels were significantly higher in *Mecp2*-null hearts at 8 weeks. These results suggest that MeCP2 deficiency affects the expression of voltage-gated channel genes in the adult heart, and that abnormal cardiac gene expression may be an arrhythmogenic substrate in *Mecp2*-null mice.

In the ventricular tissue samples, mRNA levels of *Nkx2.5*, *Tbx5*, *Myh7*, and *Kcnj12* were significantly higher in the *Mecp2*-null group (Fig. 6b), whereas *Cacna1g*, *Hcn2*, *Hcn4*, *Kcnq1*, *Cx43*, and *Cx40* mRNA levels were significantly lower in *Mecp2*-null ventricles. These observations suggest that cardiac expression of *Tbx5*, *Myh7*, *Cacna1g*, and *Hcn* genes may be regulated by MeCP2.

### DNA methylation and MeCP2 binding in cardiac genes

**Figure 7:**
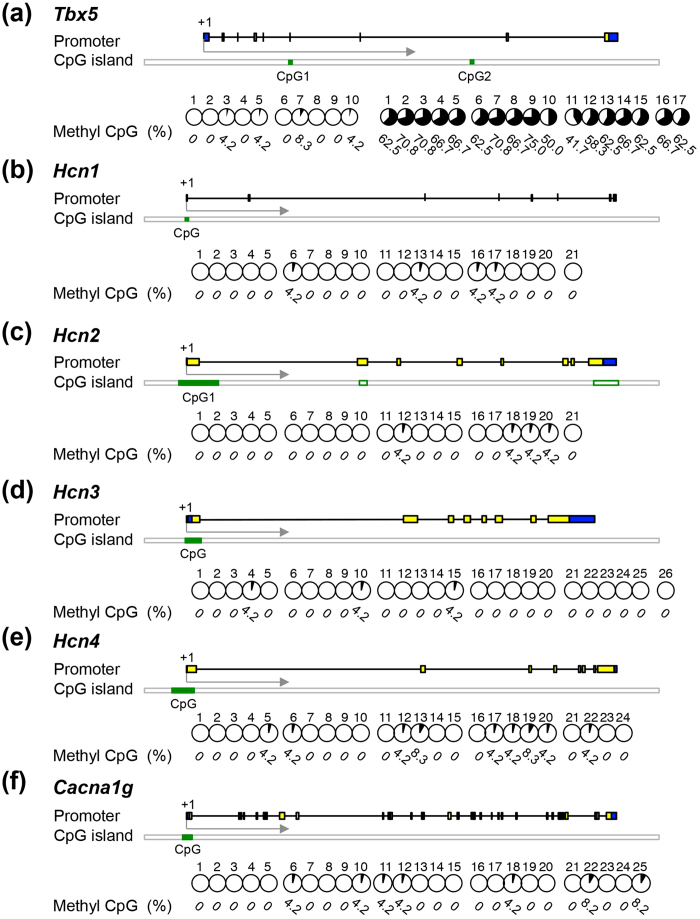
DNA methylation in potential MeCP2 target genes. Methylation of CpG islands was assessed by bisulfite sequencing in/around the following genes: *Tbx5* (**a**), *Hcn1* (**b**), *Hcn2* (**c**), *Hcn3* (**d**), *Hcn4* (**e**), and *Cacna1g* (**f**). Schematic representations of each gene are shown (not to scale). Boxes represent exons, and lines connecting boxes represent introns. Yellow boxes represent coding sequence, and blue boxes represent untranslated regions. The green filled rectangles indicate the locations of the CpGs analyzed for DNA methylation. Each circle graph represents the percentage of methylated clones (number of methylated clones among 12 clones from each of two hearts, divided by 24, multiplied by 100), and the numbers below the graphs represent percent methylated cytosine. Numbers across the top indicate specific CpG dinucleotides within the CpG island. All data were obtained from two independent adult mouse hearts.

**Figure 8:**
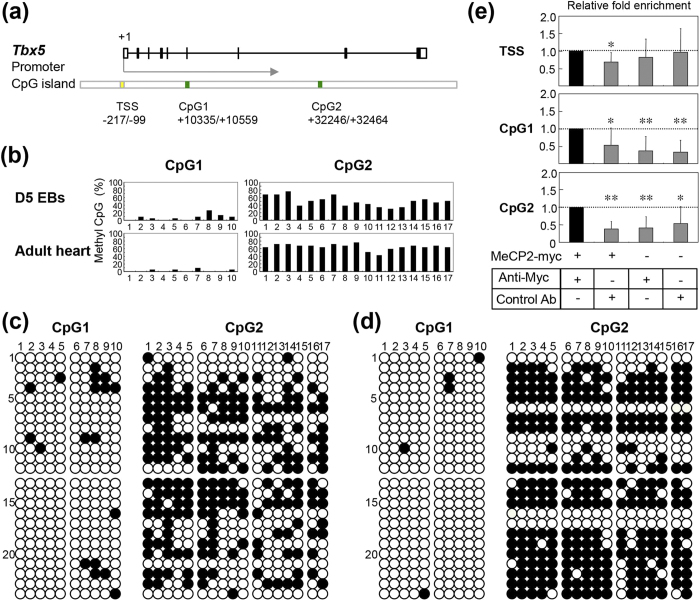
ChIP analysis and DNA methylation in the *Tbx5* gene. (**a**) Schematic representation of the *Tbx5* locus. Boxes represent exons, and lines connecting boxes represent introns. Black and white boxes represent coding sequence and untranslated region, respectively. The *Tbx5* gene contains of two CpG islands (green boxes). Numbers below each box represent the genomic location relative to the transcriptional start site (TSS, +1). (**b**) Each graph represents the percentage of methylated CpG islands (number of methylated clones among 12 clones from each of two independent EB cultures or hearts, divided by 24, multiplied by 100). (c and d) DNA methylation patterns at the *Tbx5* locus in EBs and adult hearts. Bisulfite sequencing analysis of DNA methylation; filled circles represent methylated CpG dinucleotides. Numbers across the top indicate specific CpG dinucleotides within the CpG islands. Each row represents methylation data from a single clone of two independent EB cultures (**c**) and adult hearts (**d**), indicated on the left. (**e**) MeCP2 enrichment at the *Tbx5* CpG regions. ChIP assays were performed on chromatin harvested from EBs 24 h after transient transfection with the myc-tagged MeCP2-e1 construct or pEGFP-C1 plasmid. Primers were designed to amplify DNA −217 to −99 (TSS), +10427 to +10523 (CpG1), and +32320 to +32426 (CpG2) relative to the TSS (+1). Enrichment for MeCP2 marks is shown for primer set TSS (top), CpG1 (middle), and CpG2 (bottom). Data are expressed as relative fold enrichment of anti-myc antibody (Anti-myc) in EBs transfected with myc-tagged MeCP2-e1. Data are expressed as means ± SD. ^*^p < 0.05, ^**^p < 0.01 versus Anti-myc in EBs transfected with myc-tagged MeCP2-e1, for the same primer set. As negative controls, immunoprecipitations were performed on myc-tagged MeCP2-e1 or pEGFP-C1–transfected EBs using control mouse IgG (Control Ab), and on pEGFP-C1–transfected EBs using Anti-myc.

MeCP2 regulates gene expression by binding to methylated CpG regions. Therefore, we reasoned that if MeCP2 directly regulates gene expression, the target genes might possess methylated CpG regions. First, we retrieved the genome sequence of each target gene from UCSD Genome Browser (http://genome.ucsc.edu) and identified the CpG regions in *Tbx5*, *Mef2c*, *Mesp1*, *Hcn1–4*, and *Cacna1g* (Fig. 7 and 8, Supplementary Fig. S3 and S4). To identify the CpG methylation status of the target genes, we performed bisulfite sequencing of DNA from wild-type ES cell–derived EBs on day 5, as well as from ventricular tissues from 8-week-old wild-type mice. In both normal adult heart and developing EBs, the CpG regions of Tbx5 were more heavily methylated than those of *Mesp1*, *Mef2c*, *Hcn1–4*, and *Cacna1g* (Fig. 8a–d, Supplementary Fig. S3a–d and Fig. S4a–c). These results raised the possibility that the CpG region of *Tbx5* contains a binding site for MeCP2 in developing and adult heart.

To further explore this possibility, we investigated whether MeCP2 was bound to the *Tbx5*, *Mesp1*, and *Mef2c* genes, using ChIP assays in EBs transfected with myc-tagged MeCP2-e1 (Supplementary Fig. S5). Within these genes, we found that MeCP2 was enriched in the CpG regions of *Tbx5*, but not those of *Mesp1* or *Mef2c* (Fig. 8e, Supplementary Fig. S3e and Fig. S4d). It seems likely from these experiments that MeCP2 deficiency affects *Tbx5* expression as a consequence of the loss of the interaction of MeCP2 with methylated CpG region of that gene.

## Discussion

In this study, we investigated the contribution of MeCP2 to cardiac development, structure, and function using an *in vitro* ES cell model system and an *in vivo* mouse model for RTT. The results contribute to a better understanding of the roles of MeCP2 in cardiac gene expression, development, and pathogenesis of RTT.

The ESC–EB system has been particularly useful in elucidating the molecular events involved in the specification of cell lineage differentiation. Recent studies indicated that multipotent cardiovascular progenitor cells (CPCs) become progressively lineage-restricted, and differentiate into various cardiac cell types during ESC differentiation^21^,^22^,^23^. However, the identities of the epigenetic and chromatin modifiers that regulate fate specification of CPCs remain elusive.

During cardiac differentiation of ESCs, *Mecp2*-null EBs contained a higher percentage of FLK1^+^/CXCR4^+^/PDGFRα****^+^ CPCs than wild-type EBs. Several studies have demonstrated that *Mesp1* and *Mesp2* are essential for early cardiac mesoderm formation^24^,^25^. In addition, *Mesp1* promotes the specification of CPCs during ESC differentiation^25^. These findings suggest that increased expression of *Mesp1* during *Mecp2*-null ESC differentiation may accelerate the appearance of cells co-expressing CPC markers (e.g., FLK1, CXCR4. and PDGFRa).

On the other hand, loss of MeCP2 impaired cardiac differentiation of CPCs. Further substantiating these data, the cardiac-specific markers (such as *Nkx2.5*, *Tbx5*, and *Acta2*) in isolated *Mecp2*-null CPCs were expressed lower than in wild-type CPCs, suggesting an inactivation of the cardiac differentiation gene program. Therefore, MeCP2 is involved in the development and further cardiac differentiation of CPCs during cardiac development of ESCs. These findings suggest that the disruption of normal cardiac development by MeCP2 deficiency may be one of the cardiac pathogenesis in *Mecp2*-null mice.

According to electron microscopy, IDs appeared poorly developed, i.e., immature, in *Mecp2*-null cardiomyocytes. Immunostaining for Cx43 in *Mecp2*-null ventricular tissues also revealed a significant reduction in Cx43 staining at cell–cell junctions. In addition, qRT-PCR analysis revealed lower expression of *Cx43* in *Mecp2*-null ventricular tissues. Cx43, a major component of gap junctions in the ventricle, plays a role in electrical and metabolic coupling between adjacent cardiomyocytes^26^. These results strongly indicate that impairment of intercellular communication in cardiomyocytes, due to the immaturity of ID components, is an arrhythmogenic substrate in *Mecp2*-null hearts.

In addition, we observed hypertrophied nuclei and autophagic vacuoles in *Mecp2*-null cardiomyocytes. In humans, hypertrophied nuclei are in general observed in cardiomyocytes of hypertrophied and/or failing hearts^27^. On the other hand, autophagy has been implicated in several cardiac pathologies including myocardial hypertrophy, cardiomyopathies, and ischemic heart disease^28^,^29^. These observations suggest that MeCP2 deficiency caused cardiac pathological stress, which may have contributed to activation of autophagy in the *Mecp2*-null heart.

In our *in vivo* physiological analysis, we were unable to detect obvious functional differences between wild-type and *Mecp2*-null mice, even though we did observe significant differences in ID structures and Cx43 expression. In this regard, previous studies showed that large alterations of Cx43 expression and distribution in IDs were required in order to significantly affect cardiac conduction, arrhythmogenesis, and electromechanical coupling^26^,^30^,^31^,^32^,^33^. On the other hand, alteration of Cx43 expression and distribution appears to create an arrhythmogenic substrate at less severe levels of gap-junction disorganization^26^. Indeed, heterozygous knockout of Cx43 was sufficient to increase the incidence, frequency, and duration of ventricular tachycardias in a mouse model of acute infarction model^26^,^34^. Thus, we speculate that the change in ID structures and Cx43 patterns in *Mecp2*-null hearts may be arrhythmic co-substrates in these animals, rather than leading to arrhythmia and hypertrophy per se.

On the other hand, a recent study demonstrated that *Mecp2*-null mice exhibit abnormal electrocardiographic patterns such as a prolonged QT interval and QRS complex^17^. Such discrepancies between previous studies and our findings may be due to differences in experimental systems. For instance, the *Mecp2*-null mice (C57BL/6 background) used in our study were substantially underweight starting at 4 weeks. By contrast, *Mecp2*-null mice crossed to the 129 strain were same weight as their wild-type littermates until 8 weeks of age^9^,^35^. Furthermore, subsequent survivors (after 2–3 months) became significantly heavier, with an obvious increase in deposited fat^9^,^35^. In this case, obesity may have conferred an increased risk of sudden cardiac death, in association with myocardial lipid accumulation and altered electrical properties, manifested by prolongation of the QRS duration and QT interval.

Alternatively, the PQ and QRS intervals in our *Mecp2*-null mice were significantly shorter than those in wild-type mice. The exact mechanism and clinical implications of shorter PQ and QRS are unknown. The first possibility is that a change in heart rate due to autonomic dysfunction may affect the length of the PQ interval. The second possibility is that the reduction in tissue mass may affect electrical conductivity. On the other hand, we found autophagic vacuoles in *Mecp2*-null cardiomyocytes. In this regard, a short PQ interval is a frequent finding in patients with Wolff-Parkinson-White syndrome and Pompe disease^36^,^37^,^38^. For instance, Pompe disease is characterized by mild hypertrophy and conduction abnormalities. These cardiomyocytes and conduction tissue accumulate small perinuclear vacuoles, which can alter conductive properties and may promote accelerated conduction, as seen in cases with a short PQ interval^36^,^37^,^38^. These findings suggest that accumulation of autophagic vacuoles and metabolic abnormalities is related to a change in the conductive properties of *Mecp2*-null hearts.

Our qRT-PCR analysis indicated that transcript levels of several cardiac-specific genes (such as *Tbx5*, *Nkx2.5*, *Myh7*, *Cx43*, *Cx40*, *Kcnj12*, *Kcnq1*, *Cacna1g*, and *Hcn* genes) were altered in *Mecp2*-null mice hearts. These results indicated that MeCP2 directly or indirectly regulates the expressions of various genes that play roles in the heart, including the cardiomyocytes and cardiac conduction systems.

Recent studies have revealed that epigenetic regulation of cardiac genes by DNA methylation and histone acetylation plays important roles in cardiac development, function, and pathogenesis^39^,^40^,^41^. In this study, we found that the CpG islands of *Tbx5* were highly methylated in EBs and adult ventricular tissues. In addition, our ChIP analyses showed that MeCP2 binds to the CpG islands of *Tbx5*. Furthermore, we also found that expression of *Tbx5* was significantly increased in *Mecp2*-null heart. Consistent with this, overexpression of MeCP2 in the mouse heart leads to downregulation of *Tbx5*^19^. These findings suggest that expression of *Tbx5* was directly downregulated by MeCP2 through methylation of its CpG islands.

On the other hand, recent studies indicated that MeCP2 can act either as an activator or as a repressor, depending on its interacting protein partners and target genes^1^,^2^,^3^. Indeed, MeCP2 interacts with chromatin-remodeling complexes, such as HDAC1, HDAC2, Sin3a, SMRT, and N-CoR, to regulate gene expression^3^,^39^,^40^,^41^. Further analysis of the coactivators of MeCP2 in developing and adult heart will help to elucidate the gene-regulatory properties of this protein in the cardiovascular system.

On the other hand, we cannot rule out the possibility that alteration of cardiac genes expression and myocardial structures could be related to multiple factors including autonomic dysfunction, mild hypoxia, mitochondrial abnormality, and oxidative stress^35^,^42^,^43^,^44^. Hypoxia predisposes neonatal mice to arrhythmia and sudden death, in association with ECG abnormalities^45^. In addition, hypoxia at the cellular level causes aberrant gap-junction reconstruction and misexpression of ion channels, in the heart^45^,^46^. In RTT patients, recurrent apnea and breath-holding leads to significant hypoxic and oxidative stress, both of which are detrimental to many neuronal and cardiovascular functions^44^,^47^. In fact, recent studies demonstrated that the levels of oxidative stress markers are increased, and correlated to LV systolic dysfunction, in RTT patients^18^. Those findings suggested that increased oxidative stress via respiratory depression may lead to aberrant ion channel and Cx43 expression, and predisposes the animal to arrhythmia and sudden death. Thus, alteration of cardiac gene expression and the immaturity of ID components can be attributed, at least in part, to several pathological conditions in *Mecp2*-null mice.

In conclusion, loss of MeCP2 impairs the development and further cardiac differentiation of CPCs. The resultant CPC aberrations might contribute to cardiac structural abnormalities upon deletion of MeCP2. On the other hand, in the adult hearts, MeCP2 is essential for maintaining the expression of cardiac genes, which may have critical implications in the control of heart homeostasis and its adaptation to pathologic states. The two scenarios are not mutually exclusive, and both factors may contribute to cardiac aberrations in *Mecp2*-null mice. Indeed, overexpression of MeCP2 in the mouse heart leads to embryonic lethality with cardiac septum hypertrophy^19^. In addition, MeCP2 is upregulated in infarcted hearts, and knockdown of MeCP2 by specific siRNAs in infarcted hearts reduced apoptosis in the ischemic myocardium^48^. Taken together, our study reveals that MeCP2 is an important regulator of cardiac gene expression responsible for maintaining normal cardiac development and cardiomyocyte structure. In addition, loss of MeCP2 in the heart is one of the causes of cardiac dysfunction, including arrhythmias, in the *Mecp2*-null mouse model of RTT.

## Methods

### Animal

*Mecp2*^*−/flox*^ female mice (B6.129P2(C)-*Mecp2*^*tm1.1Bird*^*/J* strain) were purchased from the Jackson Laboratory (Bar Harbor, ME) and mated with wild-type C57BL/6 male mice^9^. Genotyping was performed by PCR analysis of genomic DNA according to the protocol provided by the supplier (http://jaxmice.jax.org/pub-cgi/protocols/protocols.sh?objtype=protocol&protocol_id=598). DNA samples were extracted from the tails of newborn mice after digestion with proteinase K. All experiments were performed in accordance with the National Institutes of Health Guidelines for the Care and Use of Laboratory Animals and were approved by the Animal Research Committee of Kurume University.

### Physiological analysis

Electrocardiograms (ECGs) were performed on wild-type (*Mecp2*^*flox/y*^) mice and MeCP2-null (*Mecp2*^*−/y*^) mice at the ages of 6 and 8 weeks^49^. Mice were anesthetized with chloral hydrate (400 mg/kg) until loss of the foot reflex, and then analyzed by surface lead II ECG (Power Lab/4SP with ML135 Dual Bio, AD Instruments Japan Inc., Nagoya, Japan). RR interval, PR interval, and QRS and QT durations were recorded. The QT interval was ‘corrected’ for heart rate according to the formula QTc = QT / (RR/100)^0.5^.

Transthoracic echocardiographic (ECO) studies were performed under anesthesia using a Vevo770 ultrasound machine (VisualSonics Inc., Toronto, ON, Canada) equipped with a 30-MHz probe. Mice at the age of 8 weeks were anesthetized with chloral hydrate (400 mg/kg) and subjected to echocardiography as previously described^50^. Left ventricular end-systolic and end-diastolic dimensions (LVDs and LVDd, respectively) were measured from the LV M-mode trace. Percent fractional shortening (%FS) of the LV was calculated as %FS = (LVDd – LVDs)/LVDd × 100.

### Histology

Once the physiological measurements were complete, the mice were sacrificed. Hearts were removed, weighed, and cut into transverse slices at the level of the mid-papillary muscle. The slices were then fixed in 4% paraformaldehyde, embedded in paraffin, cut into 5-μm thick sections, and stained with hematoxylin and eosin^29^.

For cryosections (5 μm), mouse hearts were equilibrated in 30% sucrose solution and mounted in FSC 22® frozen section compound. (Leica, Wetzlar, Germany). Sections were fixed with ethanol at RT for 10 min, subjected to immunofluorescence staining with primary antibodies against connexin 43 (1:500; Sigma-Aldrich) and N-cadherin (1:500 dilution, Chemicon, Temecula, CA, USA), and then visualized with secondary fluorescent antibodies. Sections were counterstained with Hoechst33342 and examined on a Zeiss Axioskop2 Plus microscopy system (Carl Zeiss, Oberkochen, Germany). Photomicrographs were captured using an AxioCam HRc digital camera.

### Transmission electron microscopy

Cardiac tissues were prepared for ultrathin sections as previously described^29^. Briefly, cardiac tissue was quickly cut into 1-mm cubes, immersion-fixed with 2.5% glutaraldehyde in 0.1 M phosphate buffer (pH 7.4) overnight at 4 °C, and postfixed in 1% buffered osmium tetroxide. The specimens were then dehydrated through a graded ethanol series and embedded in epoxy resin. Ultrathin sections (90 nm) were stained with uranyl acetate and lead citrate, and then observed under a transmission electron microscope (H-800; Hitachi High-Technologies, Tokyo, Japan).

### PCR analysis

Extraction of total RNA, RT-PCR analysis, and electrophoresis were carried out as described previously^51^,^52^. Quantitative real-time PCR was performed on a LightCycler Nano (Roche Diagnostics, Basel, Switzerland) using FastStart Essential DNA Green Master (Roche Diagnostics). Data analysis was performed using the LightCycler Nano software version 1.0 (Roche Diagnostics). mRNA expression levels were normalized against the level of Gapdh mRNA in the same sample. The evaluation of relative mRNA levels in each sample was carried out using the ΔΔC_T_ method. Data are presented as fold changes relative to the control group. Primer sequences are listed in the Supplementary Table S4.

### DNA microarray analyses

DNA microarray analyses were performed using Agilent SurePrint G3 Mouse GE Microarray kit (8 × 60 K, Agilent technologies, Waldbronn, Germany), and the obtained data were analyzed using the GeneSpring GX software (Agilent technologies). In brief, 4.8 or 12.7 μg of total RNA was obtained from 7.7 × 10^5^ cells purified by wild-type CPCs on day 4 or from 16.0 × 10^5^ *Mecp2*-null CPCs, respectively. Subsequently, 50 ng of the total RNA from each group was reverse-transcribed, and the resulting cDNA was transcribed to cyanine 3-labeled cRNA using the Low Input Quick Amp Labeling Kit (Agilent technologies).

### Cell culture

Mouse ES cells (ESCs) in which exons 3 and 4 of *Mecp2* were flanked by loxP sites, were provided by Dr. A. Bird^9^. *Mecp2*-null (*Mecp2*^*−/y*^) and control (*Mecp2*^*flox/y*^) ESCs were generated by infection with Cre-expressing or control adenoviral vectors, and maintained as previously described^52^,^53^. To initiate differentiation, 3 × 10^5^ ESCs were cultured in 10-cm low-attachment Petri dishes with DMEM containing 10% FCS and 100 μmol/L 2-ME, but no leukemia inhibitory factor (LIF), in order to generate embryoid bodies (EBs)^52^. After 3 days in suspension, the EBs were transferred into gelatin-coated 12-well tissue culture dishes at a density of 10–20 EBs per 3.5 cm^2^, and then cultured for an additional 1–9 days.

To examine cardiac differentiation of cardiovascular progenitor cells (CPCs), sorted CPCs from 4 days of ESCs differentiation were plated on OP9 stroma cells, and cultured for an additional 6 days^21^.

### Flow cytometry and cell sorting

Flow-cytometric analysis and sorting of ESCs were described previously^52^. After 4 days of ESC differentiation, cultured cells were harvested and stained with allophycocyanin (APC )-conjugated anti-FLK1 antibody (AVAS12α1, BD Biosciences, Mississauga, ON, Canada), Alexa Fluor 488–conjugated anti-CXCR4 (CD184) antibody (eBioscience, San Diego, CA, USA), and phycoerythrin (PE)-conjugated anti-PDGFR1α (CD140a) antibody (eBioscience). FLK1^+^/CXCR4^+^/PDGFR1α^+^ cells were analyzed by flow cytometry on a FACS Cant II (BD Biosciences, San Jose, CA, USA) or sorted using a FACS Aria II (BD Biosciences). Sorted FLK1^+^/CXCR4^+^ /PDGFR1α^+^ cells were co-cultured with OP9 stromal cells at a cell density of 2 × 10^3^ / well in 48-well culture dishes.

### Immunocytochemistry

Cultures were fixed with 4% paraformaldehyde for 15 min, and then permeabilized with 0.05% Triton-X 100 for 5 min. After blocking nonspecific binding sites with 10% skim milk in PBS for 1 hr, cultures were immunocytochemically stained using primary antibodies against sarcomeric myosin heavy chain (sMHC) (1:20 dilution; MF20, Developmental Studies Hybridoma Bank, University of Iowa, Iowa City, IA, USA), Nkx2.5 (1:100 dilution; Santa Cruz Biotechnology, Dallas, TX, USA), or CD31 (PECAM-1) (1:200 dilution; eBioscience), followed by secondary fluorescent antibodies as described previously^52^. Samples were counterstained with Hoechst 33342 and examined under an Olympus IX-71 microscope (Olympus Japan, Tokyo, Japan). Photomicrographs were captured using an Olympus DP70 digital camera or a Biorevo BZ-9000 fluorescence microscope (KEYENCE Co., Osaka, Japan); images were analyzed using the BZ-II application.

### Immunoblotting

Cell extracts were prepared from ESC cultures and hearts (n = 5 from each group) as described previously^29^,^53^. Western blot analysis was performed using rabbit anti-LC3 polyclonal antibody (PD014, MBL, Woburn, MA, USA), mouse anti-GAPDH (clone 6C5) monoclonal antibody (Millipore, Temecula, CA, USA), mouse anti–c-myc (clone 9E10) monoclonal antibody (Roche Diagnostics, Penzberg, Germany), and CF^TM^ 680– or CF^TM^ 770–conjugated goat anti-mouse or rabbit IgG secondary antibodies (Biotium, Hayward, CA, USA). Fluorescence emission spectra were acquired using an Odyssey CLx infrared imaging system (LI-COR Biosciences, Lincoln, NE, USA).

### Bisulfite Treatment and Sequencing

Genomic DNA was extracted from EBs on day 5, or from ventricular tissues of hearts of 8-week-old mice, using the NucleoSpin® Tissue Kit (MACHEREY-NAGEL GmbH & Co. KG, Duren, Germany). The DNA was then subjected to sodium bisulfite modification using the MethylEasy^TM^ Xceed Rapid DNA Bisulphite Modification Kit (Human Genetic Signatures Pty Ltd, North Ryde, Australia). The genome sequences of each target gene were retrieved from UCSD Genome Browser (http://genome.ucsc.edu/). Discrimination of CpG islands was based on the formal definition of CpG islands (GC content greater than 50%, length over 200 bp, and statistical expectation greater than 0.6) (Gardiner-Garden and Frommer, 1987). The selected genomic sequences were subsequently exported to the primer design software MethPrimer (http://www.urogene.org/methprimer)^54^. Primer sequences used for amplification of bisulfite-modified DNA are provided in Supplementary Table S4. Each PCR product was cloned into the pGEM-T Easy Vector using the pGEM-T Easy Vector System (Promega, Madison, WI, USA) and sequenced.

### Chromatin Immunoprecipitation (ChIP)

ChIP assays were performed as described previously with minor changes^55^,^56^. EBs were transiently transfected with 1.0 μg of pCMV-MeCP2-e1-myc, alone with 1.0 μg of pEGFP-C1 to monitor transfection efficiency. Twenty-four hours after transfection, cells were cross-linked with 1.0% formaldehyde for 10 min at room temperature, and then quenched with 125 mM glycine for 5 min. Cell pellets were washed in 10 mM Tris–HCl, pH 7.5, 10 mM NaCl, 5 mM MgCl_2_, and then resuspended in 50 mM Tris–HCl, pH 8.0, 10 mM EDTA, 1.0% SDS and protease inhibitor cocktail (Nacalai Tesque, Kyoto, Japan). Cell lysates were sonicated to an average size of ∼250 bp using a Bioruptor^®^ UCD-200 (Diagenode, Sparta, NJ, USA) for 10 cycles of 30 sec ON and 30 sec OFF at 4 °C. Immunoprecipitation was performed overnight at 4 °C with 1.0 μg of mouse anti–c-myc monoclonal antibody (clone 9E10, Roche Diagnostics) or 1.0 μg of mouse anti-HA monoclonal antibody (clone 12CA5, Roche Diagnostics) as a negative control. Immune complexes were captured by incubation for 3 h at 4 °C with 10 μl of 50% Protein G–Sepharose Beads (GE Healthcare, Piscataway, NJ, USA). DNA complexes were eluted from the beads with 200 μl of 1.0% SDS, 0.1 M NaHCO_3_ (pH 8.0), and then heated at 65 °C overnight with 100 μg of proteinase K (Wako Pure Chemical Industry, Osaka, Japan) and RNase A (Sigma-Aldrich, St. Louis, MO, USA). DNA fragments were extracted with phenol/chloroform and resuspended in 50 μl of TE (pH 8.0). For semiquantitative PCR analysis, 1 μl of DNA was used per reaction. Primers used in ChIP analysis are shown in Supplementary Table S4.

### Statistical analysis

Quantitative results are expressed as means ± standard deviations (SDs). Student’s t-test was used to compare data, with p < 0.05 considered significant.

## Additional Information

**How to cite this article**: Hara, M. *et al*. Disturbance of cardiac gene expression and cardiomyocyte structure predisposes *Mecp2*-null mice to arrhythmias. *Sci. Rep*. **5**, 11204; doi: 10.1038/srep11204 (2015).

## Supplementary Material

Supplementary Information
