# Supplementary material for: Disturbance of cardiac gene expression and cardiomyocyte structure predisposes Mecp2-null mice to arrhythmias
Source: Sci Rep. 2015 Jun 15;5:11204. doi: 10.1038/srep11204 (PMC4466896; doi:10.1038/srep11204)
Supplement: Supplementary Information [file srep11204-s1.pdf]

Supplementary Information

Disturbance of cardiac gene expression and cardiomyocyte structure predisposes *Mecp2*-null mice to arrhythmias

Munetsugu Hara, Tomoyuki Takahashi, Chiaki Mitsumasu, Sachiyo Igata, Makoto Takano, Tomoko Minami, Hideo Yasukawa, Satoko Okayama, Keiichiro Nakamura, Yasunori Okabe, Eiichiro Tanaka, Genzou Takemura, Ken-ichiro Kosai, Yushiro Yamashita, Toyojiro Matsuishi

Inventory of supplemental Items

-Supplementary Tables

Related to DNA microarray

- Table S1

Related to Figure 4:

- Table S2
- Table S3

Related to Methods

- Table S4

-Supplementary Figures and Figure Legends

Related to Figure 5:

- Supplementary Figure S1
- Supplementary Figure S2

Related to Figure 7 and 8:

- Supplementary Figure S3
- Supplementary Figure S4
- Supplementary Figure S5

Table S1.

Genes upregulated or downregulated in the purified FLK1/CXCR4/ PDGFRα -positive cardiovascular progenitor cells derived from wild-type and *Mecp2*-null ES cells.

Accession No.	Description	Fold difference
NM_009876	cyclin-dependent kinase inhibitor 1C (P57) (Cdkn1c), transcript variant 2	51.1
NM_008006	fibroblast growth factor 2 (Fgf2)	10.5
NM_053093	tachykinin 4 (Tac4)	7.4
NM_001161832	brachyury 2 (T2)	4.5
NM_008501	leukemia inhibitory factor (Lif), transcript variant 1	3.7
NM_008266	homeobox B1 (Hoxb1)	3.5
NM_008006	fibroblast growth factor 2 (Fgf2),	3.4
NM_177781	transient receptor potential cation channel, subfamily A, member 1 (Trpa1)	3.3
NM_001164171	Myosin, heavy polypeptide 6, cardiac muscle, alpha (Myh6/alphaMHC)	3.2
NM_008006	fibroblast growth factor 2 (Fgf2)	2.9
NM_009723	ATPase, Ca++ transporting, plasma membrane 2 (Atp2b2), transcript variant 1	2.9
NM_013877	calcium binding protein 5 (Cabp5)	2.4
NM_008725	natriuretic peptide type A (Nppa)	2.3
NM_007706	suppressor of cytokine signaling 2 (Socs2), transcript variant 1	2.3
NM_008589	Mesoderm posterior 2 (Mesp2)	2.2
NM_009331	transcription factor 7, T-cell specific (Tcf7)	2.2
NM_001024716	TRIO and F-actin binding protein (Triobp), transcript variant 1	2.2
ENSMUST00000090180	ens|sema domain, immunoglobulin domain (Ig), short basic domain, secreted, (semaphorin) 3G	2.2
NM_009290	wingless-related MMTV integration site 8A (Wnt8a),	2.1
NM_001039104	transient receptor potential cation channel, subfamily M, member 1 (Trpm1), transcript variant 2	2.0
NM_009314	tachykinin receptor 2 (Tacr2)	2.0
ENSMUST00000102575	dishevelled 2, dsh homolog (Drosophila)	2.0
NM_010788	methyl CpG binding protein 2 (Mecp2), transcript variant 2	0.04
NM_172632	mitogen-activated protein kinase 4 (Mapk4)	0.15
NM_007392	actin, alpha 2, smooth muscle, aorta (Acta2)	0.16

NM_028610	developmental pluripotency associated 4 (Dppa4), transcript variant 1	0.16
NM_009606	actin, alpha 1, skeletal muscle (Acta1)	0.18
NM_011532	Mus musculus T-box 1 (Tbx1), mRNA [NM_011532]	0.19
NM_011537	T-box 5 (Tbx5)	0.21
NM_018865	WNT1 inducible signaling pathway protein 1 (Wisp1)	0.21
NM_009311	tachykinin 1 (Tac1)	0.21
NM_013693	tumor necrosis factor (Tnf)	0.23
NM_175455	ankyrin repeat domain 34B (Ankrd34b)	0.24
NM_011097	paired-like homeodomain transcription factor 1 (Pitx1)	0.24
NM_011619	troponin T2, cardiac (Tnnt2), transcript variant 9	0.26
NM_027303	ankyrin repeat domain 60 (Ankrd60), transcript variant 1	0.28
NM_013468	ankyrin repeat domain 1 (cardiac muscle) (Ankrd1)	0.29
NM_172850	ankyrin repeat and MYND domain containing 1 (Ankmy1)	0.29
NM_001164248	tropomyosin 1, alpha (Tpm1), transcript variant 1	0.31
NM_080444	ankyrin repeat and SOCS box-containing 10 (Asb10)	0.32
NM_009608	actin, alpha, cardiac muscle 1 (Actc1)	0.32
NM_018865	WNT1 inducible signaling pathway protein 1 (Wisp1)	0.33
NM_009523	wingless-related MMTV integration site 4 (Wnt4)	0.33
NM_009152	sema domain, immunoglobulin domain (Ig), short basic domain, secreted, (semaphorin) 3A (Sema3a), transcript variant 1	0.33
NM_010518	insulin-like growth factor binding protein 5 (Igfbp5)	0.34
NM_010094	left right determination factor 1 (Lefty1)	0.40
NM_009393	troponin C, cardiac/slow skeletal (Tnnc1)	0.41
NM_011352	sema domain, immunoglobulin domain (Ig), and GPI membrane anchor, (semaphorin) 7A (Sema7a)	0.41
NM_001024851	ankyrin repeat domain 34A (Ankrd34a)	0.44
NM_001160012	gap junction protein, beta 3 (Gjb3), transcript variant 1	0.44
NM_080444	ankyrin repeat and SOCS box-containing 10 (Asb10)	0.44
NM_013569	potassium voltage-gated channel, subfamily H (eag-related), member 2 (Kcnh2)	0.45
NR_001461	KCNQ1 overlapping transcript 1 (Kcnq1ot1), non-coding RNA	0.46
NM_008700	NK2 transcription factor related, locus 5 (Nkx2.5)	0.46
NM_001164251	tropomyosin 1, alpha (Tpm1), transcript variant 5, mRNA	0.47
NM_145136	myocardin (Myocd), transcript variant A, mRNA	0.47
NM_009152	sema domain, immunoglobulin domain (Ig), short basic domain, secreted, (semaphorin) 3A (Sema3a), transcript variant 1, mRNA	0.48

NM_027480	ankyrin repeat domain 24 (Ankrd24), mRNA	0.49
NM_016700	mitogen-activated protein kinase 8 (Mapk8)	0.49
NM_001008231	dishevelled associated activator of morphogenesis 2 (Daam2), mRNA	0.49
NM_146024	ankyrin repeat domain 40 (Ankrd40), transcript variant 2, mRNA	0.50
NM_008711	noggin (Nog), mRNA	0.50
NM_010612	Kinase insert domain protein receptor (KDR/Flk1)	1.62
NM_011058	Platelet derived growth factor receptor, alpha polypeptide (PDGFRa)	1.03
NM_009911	Chemokine (C-X-C motif) receptor 4 (CXCR4)	0.95
NM_010136	Eomesodermin (Eomes)	1.07
NM_009309	Brachyury (T/Bra)	1.54
NM_008588	Mesoderm posterior 1 (Mesp1)	1.73
NM_008092	GATA binding protein 4 (GATA4)	0.72
NM_025282	Myocyte enhancer factor 2C (MEF2c)	0.59
NM_021459	ISL1 transcription factor, LIM/homeodomain (Isl1)	1.15
NM_007989	Forkhead box H1 (Foxh1)	1.00
NM_008213	Heart and neural crest derivatives expressed transcript 1 (Hand1)	1.51
NM_010402	Heart and neural crest derivatives expressed transcript 2 (Hand2)	1.55
NM_080728	Myosin, heavy polypeptide 7, cardiac muscle, beta (Myh7/betaMHC)	1.08
NM_001032378	Platelet/endothelial cell adhesion molecule 1 (Pecam1/CD31)	0.74
NM_016791	Nuclear factor of activated T-cells, cytoplasmic, calcineurin-dependent 1 (NFATc1)	1.12

Table S2.

Electrocardiographic parameters in wild-type and *Mecp2*-null mice

Electrocardiography was performed on *Mecp2*-null (*Mecp2*$^{-/y}$), wild-type (*Mecp2*$^{flox/y}$), and normal C57BL/6 male (BL6) mice at the age of 6 or 8 weeks. RR, time interval between two consecutive R waves; PQ, interval between the start of P and Q waves; QRS, interval from beginning of Q wave to the end of S wave; QT, interval from beginning of Q wave to end of T wave; QTc, QT interval corrected for heart rate, $QTc = QT / (RR/100)^{0.5}$. The T wave in mice ends when it returns to the isoelectric baseline. Significant differences in these parameters are indicated as follows: *$p < 0.05$ and **$p < 0.01$ for BL6 vs. *Mecp2*$^{-/y}$; †$p < 0.05$ and ††$p < 0.01$ for BL6 vs. *Mecp2*$^{flox/y}$; ‡$p < 0.05$ and ‡‡$p < 0.01$ for *Mecp2*$^{-/y}$ vs. *Mecp2*$^{flox/y}$ mice. All comparisons were evaluated using Student's t-test.

6 weeks	RR (msec)	PQ (msec)	QRS (msec)	QT (msec)	QTc (msec)	n
BL6	126 ± 15	40 ± 2	10 ± 2	46.9 ± 4.2	42.1 ± 3.2	11
Mecp2$^{flox/y}$	109 ± 9 ††	37 ± 3 ††	10 ± 1	44.2 ± 3.8	41.7 ± 2.4	12
Mecp2$^{-/y}$	106 ± 9 **	33 ± 3 **, ‡	9 ± 1 ‡‡	42.3 ± 4.6	41.2 ± 4.1	14
**8 weeks**	**RR (msec)**	**PQ (msec)**	**QRS (msec)**	**QT (msec)**	**QTc (msec)**	**n**
BL6	115 ± 10	38 ± 3	12 ± 3	45.1 ± 2.5	42.1 ± 3.2	10
Mecp2$^{flox/y}$	115 ± 18	37 ± 2	10 ± 1	45.6 ± 6.4	41.7 ± 2.4	11
Mecp2$^{-/y}$	110 ± 8	33 ± 2 **, ‡‡	9 ± 1 **, ‡‡	43.8 ± 3.9	41.2 ± 4.1	10

Table S3.

Echocardiographic Parameters in wild-type and *Mecp2*-null mice.

Echocardiography was performed on *Mecp2*-null (*Mecp2*$^{-/y}$), wild-type (*Mecp2*$^{flox/y}$), and normal C57BL/6 male (BL6) mice at the age of 8 weeks. Left ventricular end-systolic and end-diastolic dimensions (LVDs and LVDd, respectively) were measured from the LV M-mode trace. Percent fractional shortening (%FS) of the LV was calculated as %FS = (LVDd − LVDs)/LVDd × 100. Significant differences in these parameters are indicated as follows: $^{*}p < 0.05$ and $^{**}p < 0.01$ for BL6 vs. *Mecp2*$^{-/y}$; ‡$p < 0.05$ and ‡‡$p < 0.01$ for *Mecp2*$^{-/y}$ vs. *Mecp2*$^{flox/y}$. All comparisons were evaluated using Student's t-test.

8 weeks	IVST (mm)	PWT (mm)	LVDd (mm)	LVDs (mm)	%FS (%)	n
BL6	0.72 ± 0.02	0.73 ± 0.03	3.39 ± 0.15	2.01 ± 0.16	40.9 ± 2.94	11
Mecp2$^{flox/y}$	0.72 ± 0.03	0.74 ± 0.02	3.37 ± 0.16	2.03 ± 0.17	39.7 ± 3.40	9
Mecp2$^{-/y}$	0.56 ± 0.04 **, ‡‡	0.56 ± 0.05 **, ‡‡	2.70 ± 0.17 **, ‡‡	1.55 ± 0.12 **, ‡‡	42.6 ± 2.31 ‡	12

Table S4. Primers used for PCR, quantitative real-time PCR, ChIP, and bisulfite sequencing analysis.

**PCR and quantitative real time PCR**			
Gene	Forward Primer	Reverse Primer	Ta (°C)
Mecp2	5'-GGTAAAACCCGTCCGGAAAATG-3'	5'-TTCAGTGGCTTGTCTCTGAG-3'	61
Mesp1	5'-GTCTGCAGCGGGGTGTCGTG-3'	5'-CGGCGGCGTCCAGGTTTCTA-3'	61
Mesp2	5'-CGCCTGGCCATCCGCTACAT-3'	5'-ACCCCCAGGACACCCCACTACT-3'	61
T	5'-ATCAAGGAAGGCTTTAGCAAATGGG-3'	5'-GAACCTCGGATTCACATCGTGAGA-3'	60
Eomes	5'-TGTTTTCGTGGAAGTGGTTCTGGC-3'	5'-AGGTCTGAGTCTTGGAAGGTTCATTC-3'	60
Tbx5	5'-GCTGATAACAAATGGTCCGTA-3'	5'-CCACAGGATACTCTTTACTTTGC-3'	61
Nkx2.5	5'-AAGCAACAGCGGTACCTGTC-3'	5'-GCTGTCGCTTGCACTTGTAG-3'	59
Gata4	5'-CTGTCATCTCACTATGGGCA-3'	5'-CCAAGTCCGAGCAGGAATTT-3'	57
Mef2c	5'-AGATCTGACATCCGGTGCAG-3'	5'-TCTTGTTCAGGTTACCAGGT-3'	55
Islet1	5'-CCACAAGCAGCCGGAGAAGAC-3'	5'-GAGGGTTGGCGGCATAGCAG-3'	63
Flk1	5'-GGCGGTGGTGACAGTATCTT-3'	5'-CTCGGTGATGTACACGATGC-3'	57
Cxcr4	5'-GTGACCGCCTTTACCCCGATAGC-3'	5'-TGACCCCCAAAAGGATGAAGGAGT-3'	60
Pdgfra	5'-CTGGTGCCTGCCTCCTATGAC-3'	5'-CACGATCGTTTCTCCTGCCTTAT-3'	60
Acta2	5'-GAGAAGCCCAGCCAGTCG-3'	5'-CTCTTGCTCTGGGCTTCA-3'	58
Pecam1	5'-GTCATGGCCATGGTCGAGTA-3'	5'-TCCTCGGCGATCTTGCTGAA-3'	57
Nfatc1	5'-CCTGACCACCGATAGCAC-3'	5'-GCTCGTATGGACCAGAATGT-3'	57
Myh6	5'-ATCATTCCCAACGAGCGAAAG-3'	5'-AAGTCCCCATAGAGAATGCGG-3'	59
Myh7	5'-TACCTCATGGGGCTGAACTC-3'	5'-CCCTTGGTGACGTACTCGTT-3'	57
Myl7	5'-TGACCCAGGCAGACAAGTTC-3'	5'-CGTGGGTGATGATGTAGCAG-3'	60
Myl2	5'-AAGGTGTTTGATCCCGAGGG-3'	5'-GGGAAAGGCTGCGAACATCT-3'	60
Nppa	5'-CGGTGTCCAACACAGATCTG-3'	5'-TCTCTCGAGGTGGGTTGAC-3'	58
Ank2	5'-AGATTACTGTGCAGCATAACAGG-3'	5'-TGGTTGTAAAGGAAACACACTCA-3'	59
Scn5a	5'-GCAGAAGGTGAAGTTCGTGG-3'	5'-TGAAGACCAAGTTTCCGACC-3'	60
Kcnq1	5'-CATCGGTGCCCGTCTGAACAGG-3'	5'-TTGCTGGGTAGGAAGAGCTCAG-3'	59
Kcnh2	5'-GATCGCCTTCTACCGGAAA-3'	5'-CATTCTTCACGGGTACCACA-3'	56
Kcne1	5'-CCCAATTCCACGACTGTTCT-3'	5'-CAGCTTCTTGGATCGGATGT-3'	59
Kcne2	5'-CTGGAGGATGCCTTCAAAAA-3'	5'-TGCCAATCTTCCACGATGTA-3'	55
Kcnj2	5'-TTGGCTGTGTGTTTTGGTTGAT-3'	5'-AGTCTGCGTTGTCAATGTCCTG-3'	60
Kcnj12	5'-ATCATCTTCTGGGTCATTGCTGTC-3'	5'-CGTCTCGAGGTCCTGACGGCTAAT-3'	65
Cacna1c	5'-CCCTTCTTGTGCTCTTCGTC-3'	5'-ACACCCAGGGCAACTCATAG-3'	60
Cacna1d	5'-ACATTCTGAACATGGTCTTCACAG-3'	5'-AGGACTTGATGAAGGTCCACAG-3'	58
Cacna1g	5'-TCCTTGGAGATGCTGCTGAAG-3'	5'-CACACGCTGATGACCACAATG-3'	58
Hcn1	5'-CTCTTTTTGCTAACGCCGAT-3'	5'-CATTGAAATTGTCCACCGAA-3'	55
Hcn2	5'-TTTCAACGAGGTGCTGGAGG-3'	5'-TGGCATTCTCCTGGTTGTTG-3'	61
Hcn3	5'-GACACCCGCCTCACTGATGGAT-3'	5'-GTTTCCGCTGCAGTATCGAATTC-3'	61
Hcn4	5'-CGACAGCGCATCCATGACTA-3'	5'-GCTGGAAGACCTCGAAACGC-3'	58
Cx40	5'-CAGAGCCTGAAGAAGCCAAC-3'	5'-CGAGCCTTCCACGTTAGAG-3'	60
Cx43	5'-GAGAGCCCGAACTCTCCTTT-3'	5'-TGGGCACCTCTCTTTCACTT-3'	60
Gapdh	5'-CCAGAACATCATCCCTGCATC-3'	5'-CCTGCTTCACCACCTTCTTGA-3'	57

**ChIP analysis**		
Primer name	Sequence	Ta (°C)
Mef2c CpG-F1775<br>Mef2c CpG-R1864	ATCCTGGATCGTGGCTTTTA<br>CGCCCCCTTTAGATTCTCAG	59
Mef2c TSS-F<br>Mef2c TSS-R	TGAGGAGGGAGCTGCAGTAT<br>CCGTTTCTCTATCCCAACCA	60
Tbx5 TSS-F4798<br>Tbx5 TSS-R4906	CTGGCTCAGAGCAGTCAACA<br>CCCCAAACTCTTGAACCACT	59
Tbx5 CpG1-F<br>Tbx5 CpG1-R	CGCTGTGACTTCGTACCAGA<br>CATGCGTCCACATAAAGCAG	59
Tbx5 CpG2-F<br>Tbx5 CpG2-R	GCTGCAGTGTAGAGGGAAGG<br>CACGGGTCTGGGTTGTAAA	59
Mesp1 Tbx-F<br>Mesp1 Tbx-R	TGGGTTCCTTTGGGAGCTGCTTGG<br>CTCTCACCTCTGTTCTGATGGGG	62
Mesp1 TSS-F<br>Mesp1 TSS-R	TTGGGTCCGCTGTAGCTTTATC<br>GCTATGGTTCAAAGGGTTCAGGC	62
Mesp1 CpG-F462<br>Mesp1 CpG-R552	GAGGTTGTCCTCGCTGAGTC<br>CCAGAACCTGACCAAGATCG	60

**Bisulfite sequencing analysis**	Sequence	Sequence size
Meth Mef2c-F<br>Meth Mef2c-R	GGTTAAAAGTTTTATATATTTTGGG<br>CTAACAAAAAAATCCTTCTAAAATC	243 bp, CpG in product:17
Meth Mesp1-F<br>Meth Mesp1-R	GATTTTGGTTAGGTTTTGGT<br>AACAACCCTACACCACCCTAC	152 bp, CpG in product:28
Meth Tbx5 CpG1-F<br>Meth Tbx5 CpG1-R	AAGGTAAGTTGAGAGAGTTTTTATT<br>CTATAAAACTATAAACCCACCCCTC	133 bp, CpG in product:10
Meth Tbx5 CpG2-F<br>Meth Tbx5 CpG2-R	GTTAGAAATTGGGTAAAGTATTTGT<br>AACTCCCTAACATTCTATTCCCTAC	268 bp, CpG in product:17
Meth HCN1-F<br>Meth HCN1-R	GTTGGGTAGAAGTGGTATAGG<br>CCTAATACTAAAACTAAAACTAAAACTAC	157 bp, CpG in product:21
Meth HCN2-F<br>Meth HCN2-R	GGGGTTTGGATTATTTATTTTTATAG<br>CCTCTACACACCAAAATCCCTC	217 bp, CpG in product:21
Meth HCN3-F<br>Meth HCN3-R	GATTTTTATTGTTTTGTGGTTGT<br>CTAAACTACTCTCCCAAAAACTTAC	294 bp, CpG in product:26
Meth HCN4-F<br>Meth HCN4-R	TTTTAAGAAGTTTAGGTAGGTAGGT<br>CTCCTCTAACTAAACCCAACC	222 bp, CpG in product:24
Meth Cacna1g-F<br>Meth Cacna1g-R	TTTTTTGTTTTTAGGGGTATGTTGT<br>AACCCCTTAAATCCTACTCCAACTA	299 bp, CpG in product:25

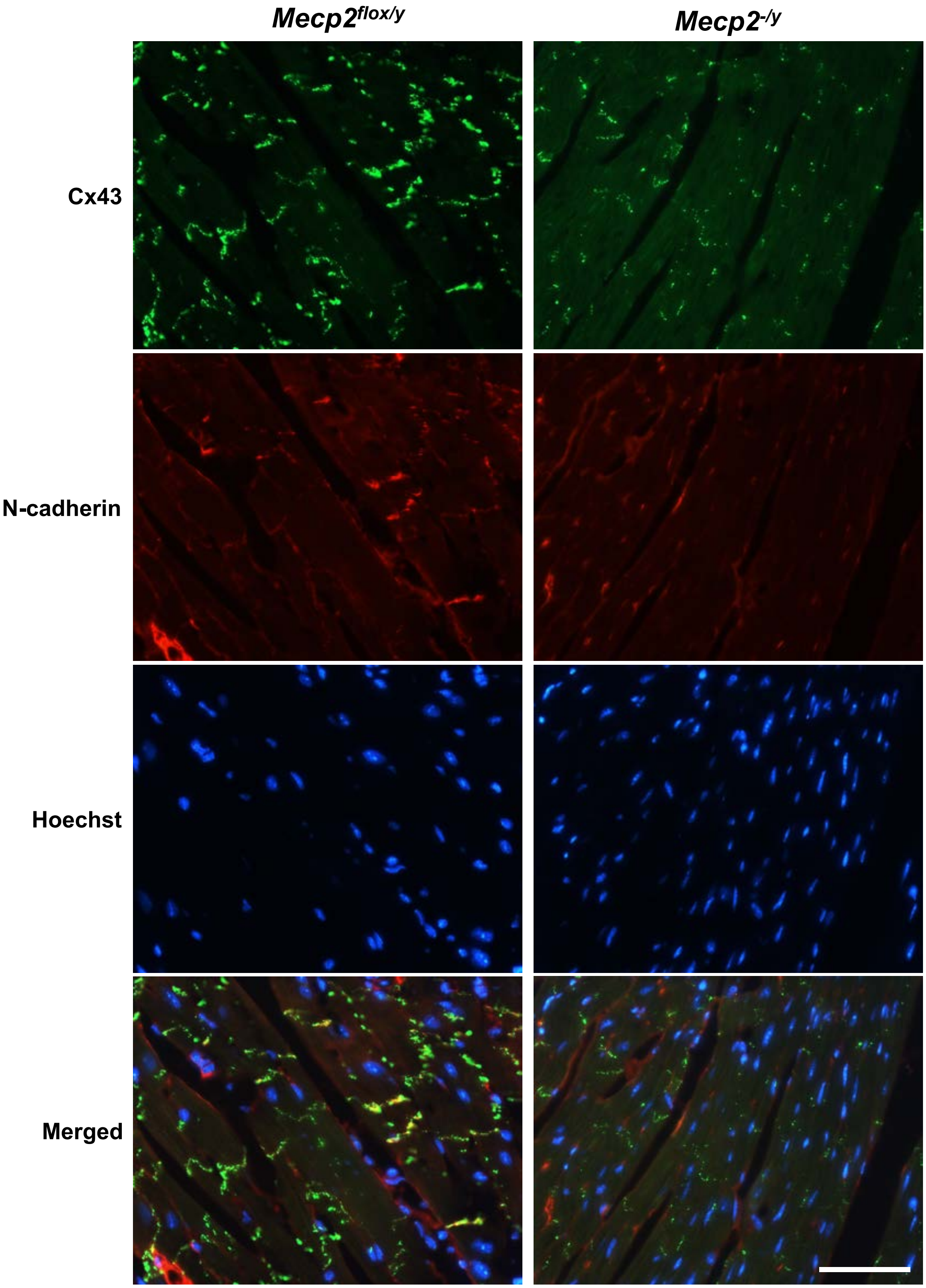

Figure S1.

Representative immunofluorescence images showing the distribution of Cx43 signal in wild-type and *Mecp2*-null left ventricular tissues. Cryosections of left ventricular tissue obtained from 8-week-old wild-type (left panels) and *Mecp2*-null (right panels) mice were stained for Cx43 (green, upper panels) and N-cadherin (red, upper-middle panels), Hoechst 33342 (blue, lower-middle panels), and merged images (bottom panels). There was an evident change in the pattern of Cx43 staining in *Mecp2*-null hearts, relative to that in wild-type hearts. Scale bars indicate 50 μm.

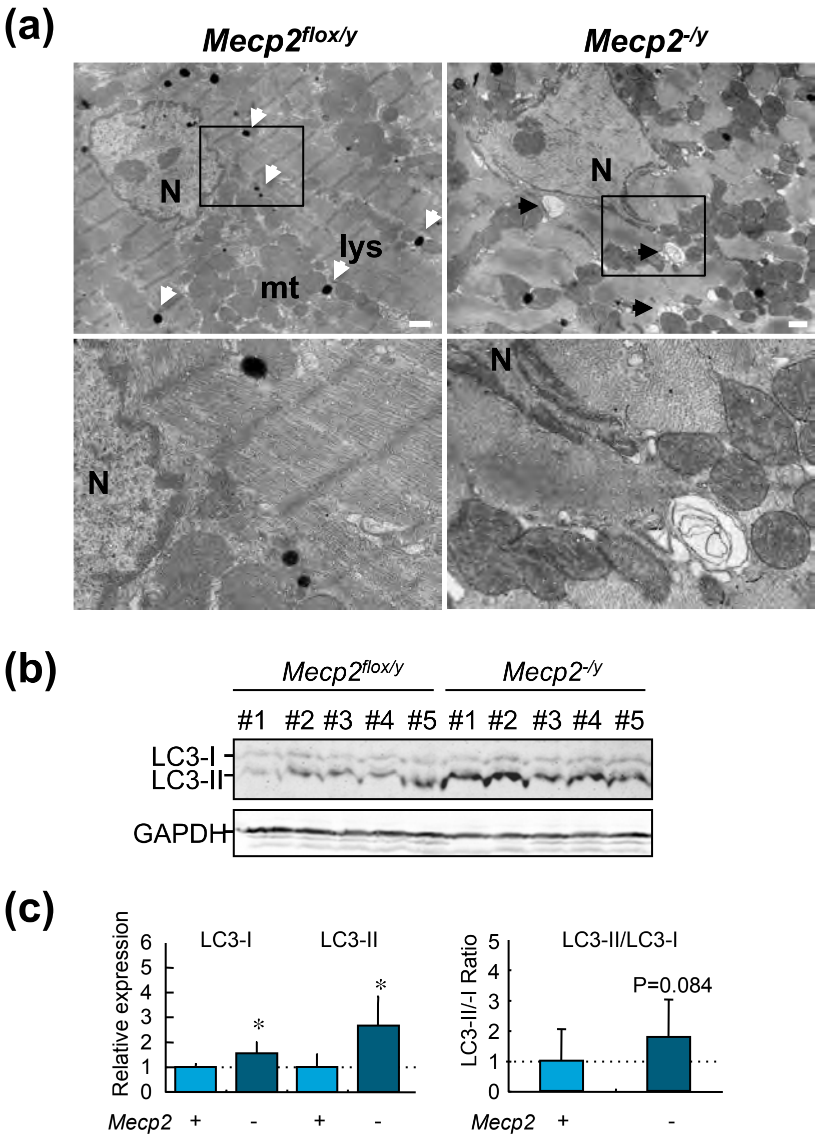

Figure S2.

Transmission electron microscopy of hearts from *Mecp2*-null mice.

(a) Transmission electron microscopy analysis of left ventricular tissues in wild-type (left panells) and *Mecp2*-null mice (right panels) hearts. Electron micrographs show more remarkably hypertrophied nucleus and more abundant autophagic vacuoles (black arrows) in contrast to less abundant lysosomes (Lys, white arrows) suggesting autophagy activation in cardiomyocytes from *Mecp2*-null mice. The area in the rectangle is shown at higher magnification in the lower panels. N, nucleus; mt, mitochondria. Scale bars indicate 1.0 µm. **(b)** Western blot performed on ventricular tissue samples from 8-week-old wild-type and *Mecp2*-null mice. Blots were probed with antibodies against LC3 and GAPDH. Blots were scanned with the Odyssey CLx infrared imaging system (LI-COR), and the band intensities were quantitated using the Image Studio software (LI-COR). **(c)** Graphs show the relative intensity of indicated protein bands (left panel) and the LC3-II/LC3-I ratio (right panel). The intensity of each protein band was normalized against that of the corresponding GAPDH band. All data were further normalized against the corresponding value from wild-type mice, which was defined as 1.0. *$p < 0.05$ versus wild-type ventricular tissue samples (n = 5, each group).

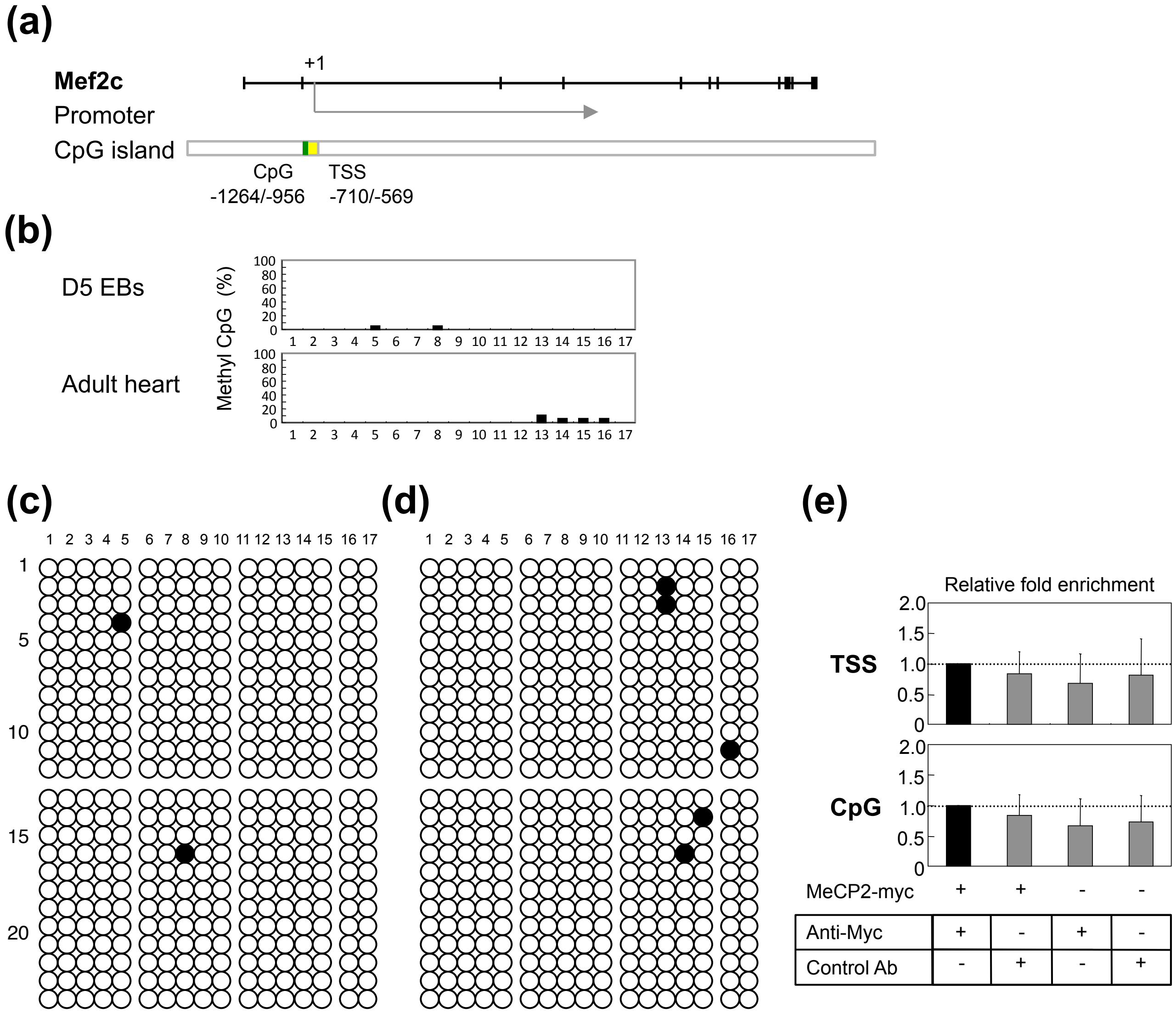

Figure S3.

ChIP analysis and DNA methylation in the *Mef2c* gene.

(a) Schematic representation of the *Mef2c* locus. Boxes represent exons, and lines connecting boxes represent introns. Black boxes represent coding sequences, whereas white boxes represent untranslated regions. The CpG island is shown as a green rectangle. The yellow box indicates the location of TSS. Numbers below each box represent the genomic location relative to the TSS (+1). **(b)** Percentage of methylated CpG islands from day 5 EBs and adult hearts. Each graph represents the percentage of methylated clones (number of methylated clones among 12 clones from each of two independent EB cultures or hearts, divided by 24, multiplied by 100). **(c** and **d)** DNA methylation patterns at the *Mef2c* locus in EBs and adult hearts. Bisulfite sequencing analysis of DNA methylation; filled circles represent methylated CpG dinucleotides. Numbers across the top indicate specific CpG dinucleotides within the CpG islands. Each row represents methylation data from a single clone of two independent EB cultures (**c**) or adult hearts (**d**), indicated on the left. **(e)** ChIP analysis was performed to investigate MeCP2 enrichment at the *Mef2c* CpG and TSS regions. ChIP assays were performed with chromatin harvested from EBs 24 h after transient transfection with the myc-tagged MeCP2-e1 construct or pEGFP-C1 plasmid. Primers were designed that amplify DNA -1226 to -1137 (CpG) and -710 to -569 (TSS) relative to TSS (+1). Enrichment for MeCP2 marks is shown for primer sets TSS (top) and CpG (bottom). Data are expressed as relative fold enrichment of anti-myc antibody (Anti-myc) in EBs transfected with myc-tagged MeCP2-e1. Data are expressed as means ± SD. $*p < 0.05$, $**p < 0.01$ versus anti-myc antibody (Anti-myc) in EBs transfected with myc-tagged MeCP2-e1, for the same primer set. Control Ab, negative control antibody. As negative controls, immunoprecipitations were performed on myc-tagged MeCP2-e1 or pEGFP-C1–transfected EBs using control mouse IgG, and on pEGFP-C1–transfected EBs using anti-myc antibody.

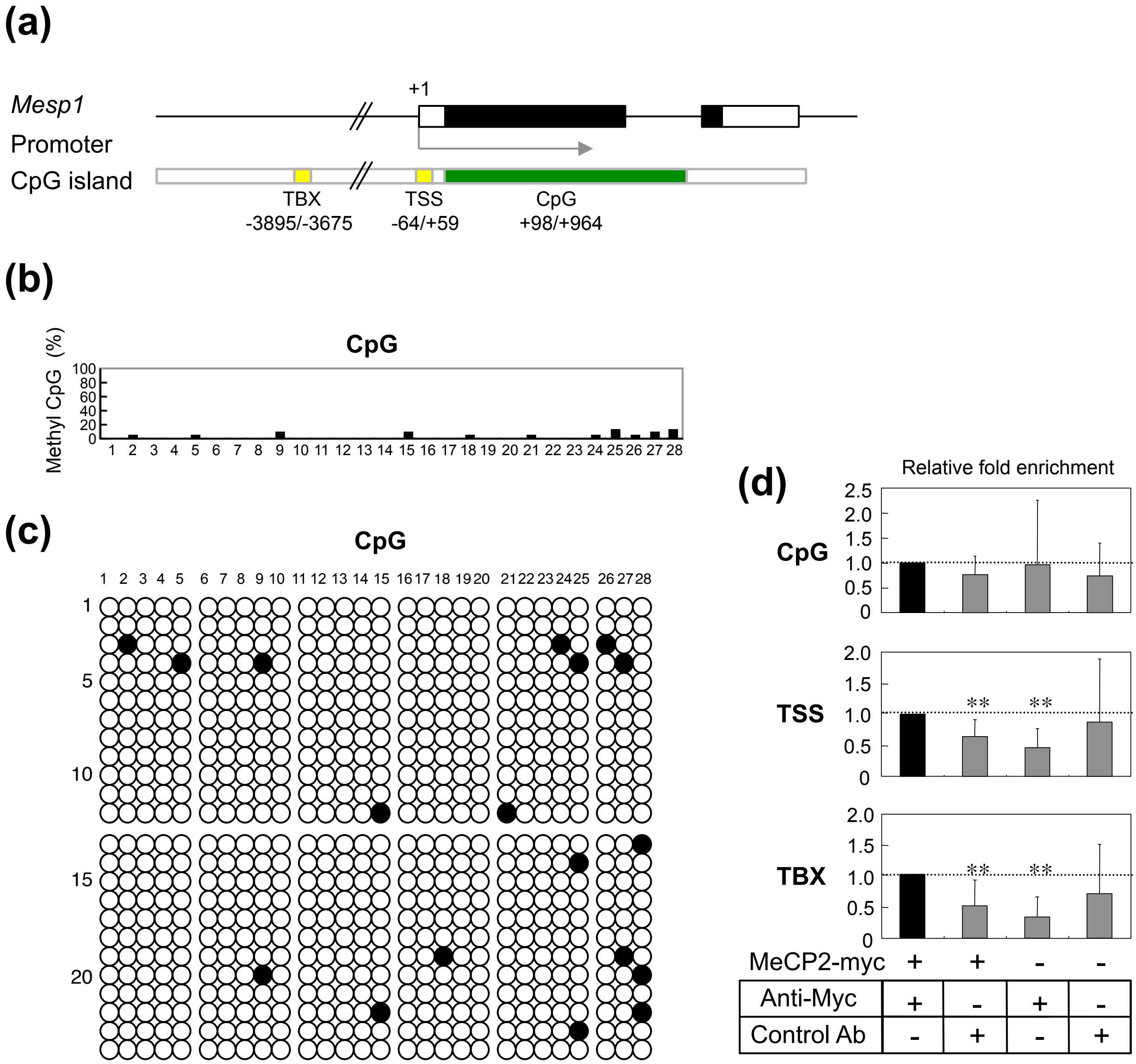

Figure S4.

ChIP analysis and DNA methylation in the *Mesp1* gene.

(a) Schematic representation of the *Mesp1* locus. Boxes represent exons, and lines connecting boxes represent introns. Black boxes represent coding sequences, whereas white boxes represent untranslated regions. The yellow boxes indicate the location of TSS and T-box binding site (TBX). Numbers below each box represent the genomic location relative to TSS (+1). **(b)** Percentage of methylated CpG islands in day 5 EBs. Each graph represents the percentage of methylated clones (number of methylated clones among 12 clones from each of two independent EB cultures, divided by 24, multiplied by 100). **(c)** DNA methylation patterns at the *Mesp1* locus in day 5 EBs. Bisulfite sequencing analysis of DNA methylation; filled circles represent methylated CpG dinucleotides. Numbers across the top indicate specific CpG dinucleotides within the CpG islands. Each row represents methylation data from a single clone of two independent EB cultures, indicated on the left. **(d)** ChIP analysis was performed to investigate MeCP2 enrichment at the *Mesp1* TSS, TBX, and CpG regions. ChIP assays were performed on chromatin harvested from EBs 24 h after transient transfection with the myc-tagged MeCP2-e1 construct or pEGFP-C1 plasmid. Primers were designed to amplify DNA -3895 to -3675 (TBX), -64 to +59 (TSS), and +411 to +501 (CpG) relative to the TSS (+1). Enrichment for MeCP2 marks is shown for primer sets CpG (top), TSS (middle), and TBX (bottom). Data are expressed as relative fold enrichment of anti-myc antibody (Anti-myc) in EBs transfected with myc-tagged MeCP2-e1. Data are expressed as means ± SD. $^*p < 0.05$, $^{**}p < 0.01$ versus anti-myc antibody (Anti-myc) in EBs transfected with myc-tagged MeCP2-e1, for the same primer set. Control Ab, negative control antibody. As negative controls, immunoprecipitations were performed on myc-tagged MeCP2-e1 or pEGFP-C1–transfected EBs using control mouse IgG, and on pEGFP-C1–transfected EBs using anti-myc antibody.

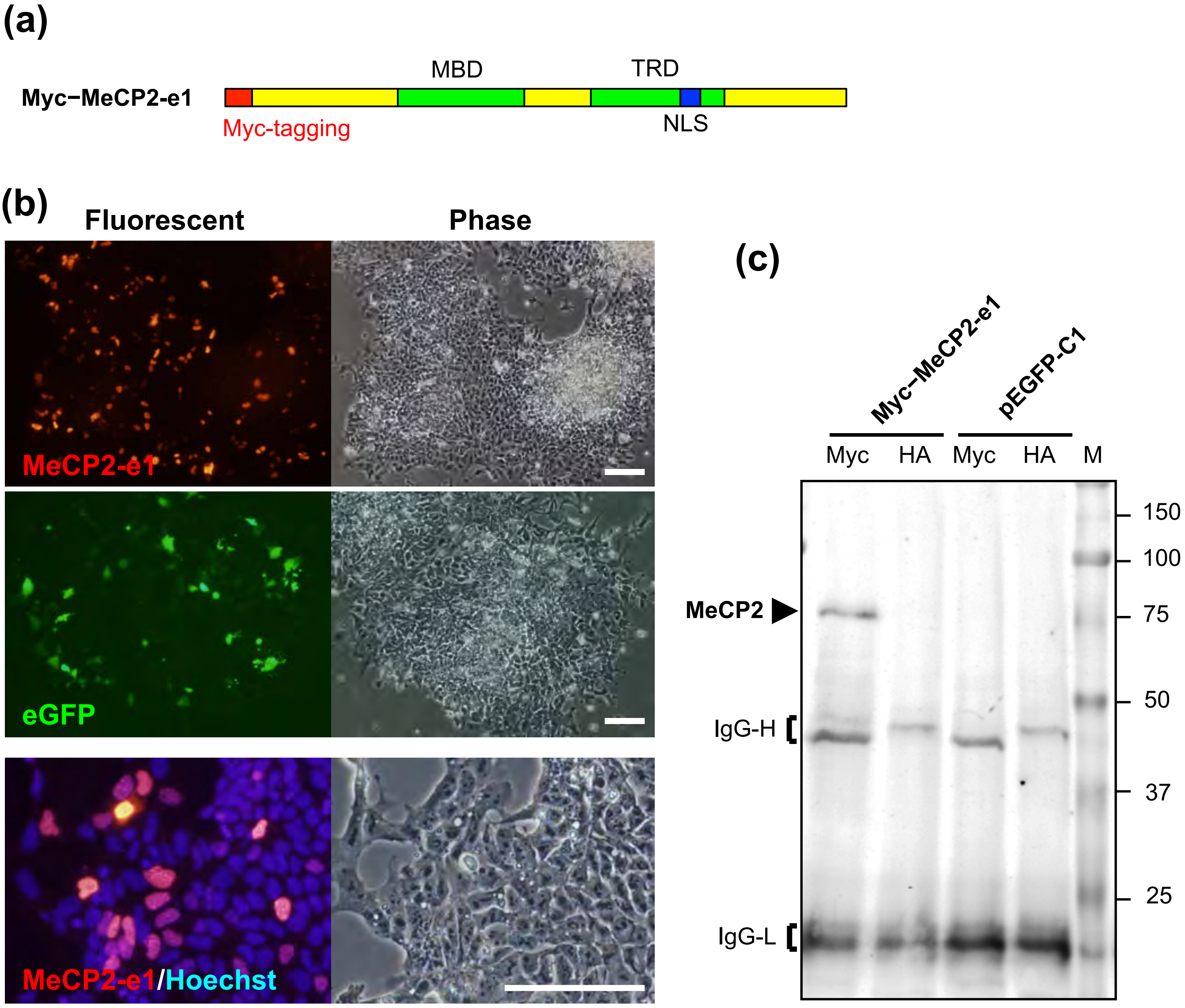

Figure S5.

Expression and immunoprecipitation of epitope-tagged MeCP2 protein.

(a) Schematic representation of the myc-MeCP2-e1 construct used in this study. **(b)** Expression of MeCP2 protein in EBs. EBs were transfected with constructs expressing myc-MeCP2-e1 (upper and bottom panels) or enhanced green fluorescent protein (middle panels). Cells were stained for ectopically expressed MeCP2 protein with anti-myc antibody, and nuclear DNA was counterstained with Hoechst 33342. For these experiments, typical transfection efficiencies were 10–15% (12.87 ± 4.12%). Scale bar, 100 µm. **(c)** Immunoprecipitation of epitope-tagged MeCP2 protein. Epitope-tagged MeCP2-e1 protein expressed in ESCs was immunoprecipitated. The immunoprecipitates were subjected to immunoblots using anti-myc antibody.
